# The positive regulatory loop of TCF4N/p65 promotes glioblastoma tumourigenesis and chemosensitivity

**DOI:** 10.1002/ctm2.1042

**Published:** 2022-09-18

**Authors:** Yaling Hu, Bo Zhang, Peihua Lu, Jingying Wang, Cheng Chen, Ying Yin, Quan Wan, Jingjing Wang, Jiantong Jiao, Xiangming Fang, Zhening Pu, Lingli Gong, Li Ji, Lingpeng Zhu, Rui Zhang, Jia Zhang, Xusheng Yang, Qing Wang, Zhaohui Huang, Jian Zou

**Affiliations:** ^1^ Department of Laboratory Medicine The Affiliated Wuxi People's Hospital of Nanjing Medical University Wuxi Jiangsu China; ^2^ Center of Clinical Research The Affiliated Wuxi People's Hospital of Nanjing Medical University Wuxi Jiangsu China; ^3^ Center for Translational Medicine Jiangnan University Wuxi Jiangsu China; ^4^ Department of Medical Oncology The Affiliated Wuxi People's Hospital of Nanjing Medical University Wuxi Jiangsu China; ^5^ Clinical Laboratory Taixing People's Hospital Taizhou Jiangsu China; ^6^ Department of Neurosurgery The Affiliated Wuxi Second People's Hospital of Nanjing Medical University Wuxi Jiangsu China; ^7^ Department of Neurosurgery The Affiliated Wuxi People's Hospital of Nanjing Medical University Wuxi Jiangsu China; ^8^ Department of Radiology The Affiliated Wuxi People's Hospital of Nanjing Medical University Wuxi Jiangsu China; ^9^ Wuxi Cancer Institute Affiliated Hospital of Jiangnan University Wuxi Jiangsu China

**Keywords:** chemosensitivity, nuclear translocation, p65, stability, TCF4N, tumourigenesis, ubiquitination

## Abstract

**Background:**

NF‐κB signaling is widely linked to the pathogenesis and treatment resistance in cancers. Increasing attention has been paid to its anti‐oncogenic roles, due to its key functions in cellular senescence and the senescence‐associated secretory phenotype (SASP). Therefore, thoroughly understanding the function and regulation of NF‐κB in cancers is necessary prior to the application of NF‐κB inhibitors.

**Methods:**

We established glioblastoma (GBM) cell lines expressing ectopic TCF4N, an isoform of the β‐catenin interacting transcription factor TCF7L2, and evaluated its functions in GBM tumorigenesis and chemotherapy in vitro and in vivo. In p65 knock‐out or phosphorylation mimic (S536D) cell lines, the dual role and correlation of TCF4N and NF‐κB signaling in promoting tumorigenesis and chemosensitivity was investigated by in vitro and in vivo functional experiments. RNA‐seq and computational analysis, immunoprecipitation and ubiquitination assay, minigene splicing assay and luciferase reporter assay were performed to identify the underlying mechanism of positive feedback regulation loop between TCF4N and the p65 subunit of NF‐κB. A eukaryotic cell‐penetrating peptide targeting TCF4N, 4N, was used to confirm the therapeutic significance.

**Results:**

Our results indicated that p65 subunit phosphorylation at Ser 536 (S536) and nuclear accumulation was a promising prognostic marker for GBM, and endowed the dual functions of NF‐κB in promoting tumorigenesis and chemosensitivity. p65 S536 phosphorylation and nuclear stability in GBM was regulated by TCF4N. TCF4N bound p65, induced p65 phosphorylation and nuclear translocation, inhibited its ubiquitination/degradation, and subsequently promoted NF‐κB activity. p65 S536 phosphorylation was essential for TCF4N‐led senescence‐independent SASP, GBM tumorigenesis, tumor stem‐like cell differentiation and chemosensitivity. Activation of p65 was closely connected to alterative splicing of TCF4N, a likely positive feedback regulation loop between TCF4N and p65 in GBM. 4N increased chemosensitivity, highlighting a novel anti‐cancer strategy.

**Conclusion:**

Our study defined key roles of TCF4N as a novel regulator of NF‐κB through mutual regulation with p65 and provided a new avenue for GBM inhibition.

## Graphical abstract

The positive regulatory loop of TCF4N/p65 promotes glioblastoma (GBM) tumourigenesis and chemosensitivity: TCF4N upregulates NF‐κB activity by binding and promoting p65 S536 phosphorylation, nuclear‐translocation and stability; p65 promotes alternative splicing of *TCF7L2* pre‐mRNA, resulting in an increase of TCF4N.

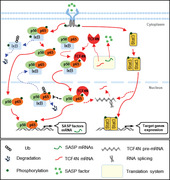

## 1 INTRODUCTION

NF‐κB has been identified as a critical factor in tumour occurrence, development and treatment resistance, serving as a useful tumour therapy target.^1^ Clinical trials with NF‐κB inhibitors, however, have failed to achieve the anticipated results.^2^ While accumulating evidence showing the tumour suppressive effects of NF‐κB,^3^, ^4^, ^5^ its oncogenic or tumour‐suppressive activities remains controversial.^6^ NF‐κB plays key roles in driving cellular senescence and cytokine production, termed senescence‐associated secretory phenotype (SASP).^6^ The dual role of cellular senescence and SASP in tumour developing and therapy response may explain the double‐edged functions of NF‐κB.^6^, ^7^, ^8^ The activation of NF‐κB is an early event and plays a critical pro‐apoptotic role in chemotherapy‐induced cytotoxicity under certain conditions and in certain cell types.^9^ Moreover, NF‐κB mediates apoptosis via cross‐talk with p53, and therapeutic response may be diminished by inhibiting NF‐κB in tumours retaining wild‐type p53.^4^, ^10^ Therefore, thoroughly understanding the function and regulation of NF‐κB in cancers is necessary prior to the application of NF‐κB inhibitors.

NF‐κB transcription factors consist of two subunits of either homo‐ or heterodimers of RelA/p65, c‐Rel and p50. These factors localise in the cytoplasm and are prevent from activating by a class of the inhibitors. Upon stimulation, the NF‐κB complex is unbound from the IκB proteins and allowed to translocate into the nucleus. However, recent findings demonstrate that the nuclear translocation of NF‐κB can also happen in the absence of stimulation, in an IκB‐dependent way.^11^, ^12^ Post‐translational modification (PTM) is also an important mechanism of NF‐κB activation regulation, which may occur without an external stimulus.^13^ Among the subunits of NF‐κB transcriptional factors, RelA/p65 is the potent transcriptional activator of the NF‐κB protein family.^12^ Multiple phosphorylation sites of p65 have been mapped, which regulate NF‐κB transcriptional activity by different mechanisms.^14^ Among these phosphorylation sites, the serine 536(S536) is the most potent site responding to inflammation‐induced phosphorylation.^15^ Phosphorylation of p65 on S536 can induce an IκB‐independent nuclear translocation that regulates distinct set of target genes, such as interleukin‐8(IL8).^16^, ^17^ p65 phosphorylation is implicated in chemotherapy‐induced cell senescence, and cooperates with p53 to promote senescence and chemosensitivity.^7^ Moreover, by introducing a phosphomimetic mutation at S536(p65/S536D), nuclear NF‐κB can overcome anticancer therapy resistance by inducing apoptosis and senescence,^18^, ^19^ further implicating S536 phosphorylation in a tumour‐suppressive role. However, S536 phosphorylation has been found no role, or even inhibitory effects on nuclear translocation and gene transcription.^20^, ^21^ Therefore, whether p65 connects or disconnects to oncogenic or anti‐oncogenic functions of NF‐κB in cancers is largely unclear.

Glioblastoma (GBM) is the most malignant human brain tumour characterised by therapy resistance and high NF‐κB activity. Recent studies suggest targeting NF‐κB/p65 as a therapeutic approach in GBM.^22^, ^23^ p65 is mostly expressed in cytoplasm with scarce nuclear localization,^24^, ^25^ indicating that the direct role of NF‐κB/p65 in gliomagenesis and chemoresistance is important. Here using tissue microarray, we identify nuclear p65 as a promising prognostic marker for GBM patients, and demonstrate the dual functions of NF‐κB in promoting tumourigenesis and chemosensitivity upon pre‐activated p65 at S536. This mechanical exploration enriches our understanding of the β‐catenin‐interacting transcription factor TCF7L2(also known as TCF4), which functions as a co‐activator of NF‐κB.^26^, ^27^, ^28^ TCF4N, an isoform of TCF7L2, binds and induces p65 S536 phosphorylation, nuclear translocation and stability, which induces a p65‐dependent, senescence‐independent SASP. The expression of TCF4N is regulated by p65‐mediated RNA alterative splicing, uncovering a positive regulatory feedback loop between TCF4N and p65 in GBM. A eukaryotic cell‐penetrating peptide targeting TCF4N further demonstrates its ability to increase chemosensitivity, highlighting a novel anti‐cancer strategy. These findings provide evidence of a positive feedback loop between TCF4N and p65 in GBM, an important step to fully understanding the role of NF‐κB in GBM.

## 2 MATERIAL AND METHODS

### 2.1 Clinical samples and immunohistochemistry

A GBM cohort was used to detect the expression of *TCF4N* mRNA. It was comprised of 50 human primary GBM tissues and 6 normal brain tissues (NB) with follow‐up data obtained from The Affiliated Wuxi People's Hospital of Nanjing Medical University and Affiliated Hospital of Jiangnan University (Data S1, Table S1). All patient materials were obtained and used in accordance with protocols approved by the institutional review boards of the participating institutions. Tissue array was performed on these 47 of 50 GBM cases and 3 normal brain tissues. Immunohistochemistry (IHC) staining was performed as described previously.^29^ Briefly, tissue slides were incubated with rabbit anti‐p65 antibody, anti‐rabbit secondary antibody (Thermo, Waltham, MA) and 3,3′‐diaminobenzidine (DAB). Slides were counterstained lightly with crystal violet. Normal rabbit IgG was applied to monitor the specificity of the IHC. p65 expression was scored according to cytoplasm and nucleus according to the established immunoreactivity scoring (IRS) system. The slides were concurrently examined and scored by two blinded pathologists. The mean IRS was considered as the final IRS (Data S1).

### 2.2 Primary cell preparation, cell lines and culture conditions

The human glioma cell lines H4, U87 and U251 were obtained from the American Type Culture Collection (ATCC). HEK293T and human breast carcinoma cell line MDA‐MB‐453 were obtained from the Cell Bank of Type Culture Collection of Chinese Academy of Science (Shanghai, China), and were cultured in DMEM with 10% fetal bovine serum (FBS; Invitrogen, Carlsbad, CA). All cell lines were validated by short tandem repeat (STR) analysis and tested for mycoplasma contamination. For patient‐derived GBM cell culture (GBM3), fresh brain GBM tissues were collected immediately after resection. After washing, mincing, and enzymatically dissociating, the derived cells were seeded in neural stem cell culture media according to previous reports.^30^ For GBM stem‐like cell (GSC) culture, GBM3 and U251 cells were seeded in a low‐attachment dish and held in the neural stem cell (NSC) culture media.^29^ Those formed neurospheres were collected for subsequent study. The first passage of neurospheres derived from U251 and GBM3 cells were dissociated and allowed to reform. To initiate GSCs differentiation, the first and second passage of spheres were cultured in media without epidermal growth factor (EGF) and basic fibroblast growth factor (bFGF).^31^ The differentiation of GSCs was identified by immunostaining and Western blot (WB) using Nestin (marker for GSCs) and GFAP (marker for GSCs differentiation) antibodies.

### 2.3 Reverse‐transcription PCR (RT‐PCR) and quantitative PCR (RT‐qPCR)

Total RNA was extracted using TRIzol reagent according to the manufacturer's instructions (Thermo). Complimentary DNA (cDNA) was synthesised according to the manufacturer's protocol (Promega, Madison, WI). To achieve the *TCF4N* fragment crossing the termination codon, PCR was performed using designed primers (Table S2). *GAPDH* served as a loading control. The products were identified by agarose gel electrophoresis and GeneGreen staining (TianGen Biotech, China). DNA fragments were isolated from the agarose gels with Gel Extraction Kit (CoWin Bioscience, China). PCR products were further sequenced. qPCR analyses were conducted to quantify mRNA expression using Taq MasterMix (CoWin Bioscience, China), with *GAPDH* as an internal control. The specific oligonucleotide primer pairs are listed in Table S3. The relative gene expression level was normalised to those of *GAPDH* and calculated using the 2^–∆∆CT^ method.^32^ For comparison of TCF4N expression between normal brains and GBM, the expression of *TCF4N* was expressed as ∆CT.

### 2.4 RNA‐seq and computational analysis

Total transcriptome RNA‐seq was performed to detect the mRNA expression profiles of U87 cells or derived xenografts expressing TCF4N at YiKe Population Health Research Institute (China) using HiSeq3000(Illumina, San Diego, CA). CASAVA v1.8 was used to align the reads to the genome, generate raw counts corresponding to each known gene (a total of 20 345 genes) and calculate the RPKM (reads per kilobase per million) values. The differential gene analysis was carried out using the DEseq2 package in the R software suite. The differential genes were selected with fold change > 1.5, and gene enrichment was analysed by Gene Set Enrichment Analysis (GSEA) method.

### 2.5 Vector construction and transduction

Full‐length cDNA encoding human *TCF4*, *TCF4N* and *RELA* (p65) were amplified by PCR and verified by DNA sequencing. The sequence of *TCF4N*, *TCF4* and *TCF4N* deletion mutant (TCF4∆N) was cloned into the lentiviral expression vector GL102(ObioTech, China) with a HA‐tag. *RELA* sequence was cloned into the plasmid GL107(ObioTech, China) with a Flag‐tag. The p65 phosphorylation mimic (S536D) was constructed based on RELA (p65) lentivirus with amino acid mutations to aspartic acid (D) at Ser536. For TCF7L2 promoter reporter vector, a 2510 bp promoter sequence of *TCF7L2*(Gene ID: 6934) covering 2000 bp upstream and 510 bp downstream of CDS was inserted into pGL4.10 vector (Promega) containing the luciferase gene.

### 2.6 CRISPR/Cas9 mediated RELA deletion and rescue

The CRISPR/Cas9 system (GeneChem) was used to establish *RELA* depleted GBM cell lines. Briefly, cells were infected with Lenti‐Cas9 lentivirus and screened by puromycin (Sigma, St. Louis, MO). The single guide RNAs (sgRNAs) for human *RELA* gene were designed and cloned into GV371 lentiviral plasmids (GeneChem). The sequence of sgRNAs and negative controls (NC) are listed in Table S4. The sgRNA containing plasmid was delivered into GBM cell lines with stable Cas9 expression by lentivirus. The Cas9/sgRNA mediated heterodimerization and digestion were assayed using the Knockout and Mutation Detection Kit (GeneChem) according to the manufacturer's protocol. For *RELA* rescue, five synonymous mutations targeting sgRNA were introduced into the new *RELA* sequence (QuikChange II XL Site‐Directed Mutagenesis Kit, Stratagene, La Jolla, CA). The new *RELA* sequence was cloned into the plasmid GL107(ObioTech) with a Flag‐tag. Western blot was performed to confirm *RELA* knockout and rescue using a p65 antibody.

### 2.7 Minigene splicing assay

TCF7L2 minigene sequence containing exon 7, 8, 9, 10 of *TCF7L2*, with 200 bp intronic segments flanking the donor and acceptor splice sites of intron 8 inserted between exon 8 and exon 9 was cloned into GV658 plasmid (GeneChem, Shanghai, China). To detect splicing alterations, TCF7L2 and TCF4N specific primers were designed (Figure 6J). They shared the forward primer in exon 7, and the specific reverse primer was located in intron 8 and exon 10 respectively. The PCR products were separated by agarose gel electrophoresis, and scanned with a scanner with GAPDH as an internal control. The primer sequences used were listed in Table S5.

### 2.8 Recombinant protein purification

The 11 amino acids of TAT‐PTD (YGRKKRRQRRR) obtained from the HIV TAT protein can deliver macromolecular cargo into a variety of cell types. A cell permeable TCF4N (named as 4N) was generated by fusing the TAT‐PTD to N‐terminal using standard molecular cloning techniques and a pcDNA3.1 vector with Flag and His tag. The plasmid was transfected into HEK293T cells and a stable cell line was produced using G418(Sigma) selection. Using the High Affinity Ni‐NTA Resin Kit protocol (Genscript, China), cellular proteins were collected and recombinant proteins were affinity purified. Eluted proteins were dialyzed against 20 mmol/L of HEPES (pH 8.0) plus 150 mmol/L of NaCl at 4°C and frozen in 10% glycerol at −80°C. A peptide containing a scrambled version of the first 12 amino acids of TCF4N was synthesised directly (Genscript, China), and served as a negative control. To enforce nuclear localization, 3× Nuclear localization sequence (NLS) was added to the C‐terminal of the control peptide.

### 2.9 Immunofluorescence staining

Immunofluorescence (IF) staining was performed as described previously.^33^ Antibodies used to determine the indicated proteins were shown in Table S6. The nuclei of cells were counterstained with Hoechst 33342(Thermo). The sections or coverslips were mounted, and examined using the Olympus BX60 light (Olympus, Center Valley, PA, USA) or a laser scanning confocal microscope (Leica Microsystems GmbH, Mannheim, Germany). The specificity of IF was confirmed by primary antiserum omission and normal mouse/rabbit serum controls. To quantify cells after IF, 10 pictures of each well were captured with a 20× objective. The imaged areas were chosen randomly from at least 6 independent experiments with two replications.

### 2.10 Western blot assays

Standard Western blot assay was used for measuring protein expression, and the antibodies used are listed in Table S6.

### 2.11 Immunoprecipitation and ubiquitination assays

Immunoprecipitation (IP) was performed as previously described.^29^ Briefly, whole‐cell lysates were precipitated by Protein A/G beads (Bimake, Houston, TX) with indicated antibodies. Precipitated products were assessed by immuno‐blot (IB) analysis using the indicated antibodies (Table S6). For the ubiquitination assay, cells were treated with MG132(Abcam, Cambridge, UK) at a final concentration of 20 μM for 4 h before harvest. The ubiquitination and degradation of p65 were determined by IP using p65 antibody followed by Western blot analysis with Ubiquitin (Ub) antibody. For p65 stability assay, cells were treated with cycloheximide (CHX, 100 μg/ml; Selleck, Houston, TX) with or without MG132(20 μM; Selleck) for the indicated hours before harvest. p65 expression was determined by Western blot.

### 2.12 Cell growth assay and colony formation assay

Cell growth was assessed by the Cell Counting Kit‐8(CCK‐8) Assay Kit (Bimake). The experiments were repeated six times. For the colony formation assay, 500–1000 cells were seeded into each well of a six‐well plate with soft agar (Agarose; Sigma) and maintained in a medium containing 10% FBS for 10–14 days. The colonies were stained with 0.1% crystal violet. The number of clones containing at least 20 cells was counted using an inverted microscope.

### 2.13 3D‐tumour spheroids assay

Tumour spheroid formation was analysed as described previously.^29^ In brief, the growth of 3D‐Spheroid cells was monitored by a microscope with a real‐time camera (EVOS® FL Auto Imaging System, Life Technologies, Carlsbad, CA, USA). For sphere growth assay, photographs of tumour spheres were taken at the indicated time points, and sphere diameter was measured to reflect the growth of spheres.

### 2.14 Limiting dilution assay

Limiting Dilution assay (LDA) was performed as described previously.^34^ Spheres derived from U251 and GBM3 were dissociated into single‐cell suspensions using Accutase Reagent (Millipore, Billerica, MA) and plated in 96‐well low attachment plates (Corning, NY) by a limiting dilution fashion at 5, 20, 50, 100, 200 cells per well in NSC culture media. Fresh medium was added every 3–4 days by removing 50% of the old medium. After 2–3 weeks, tumour spheres were examined. Clonal frequency and significance were analysed using the Extreme Limiting Dilution Analysis (http://bioinf.wehi.edu.au/software/elda/).

### 2.15 GSC division assay

For GSC division assay, the procedure was performed according to the previously described method with some modifications.^35^ Briefly, the GSC spheres derived from GBM3 were dissociated and re‐cultured in NSC medium in 1 μM BrdU (Sigma) for 7 days to form secondary spheres. Then, the secondary CSLC spheres were dissociated and synchronised through a two‐step thymidine (2 mM; Sigma) sequence to control cell division for entering secondary mitosis and paired‐cell formation. The cells were washed intensively, and seeded on ornithine‐coated coverslips in NSC medium for 6 h. The cells were fixed and permeabilised. BrdU fluorescence staining was performed according to the IF protocol (Cell signalling) with BrdU antibody. Fluorescence images were visualised using a laser scanning confocal microscope (Leica, Heidelberg, Germany). Any ambiguous segregation of BrdU was excluded from analysis.

### 2.16 EdU cell proliferation assay

Cell proliferation was also assayed by EdU incorporation method as described previously.^36^ In brief, cells were cultured on polylysine (Sigma)‐coated coverslips for 24 h followed by an incubation of EdU (Thermo) for 12 h, and fixed with 4% paraformaldehyde (PFA). The staining procedure was performed according to the manufacturer's instructions for the Click‐iT^®^EdU Cell Proliferation Assays Kit (Thermo). The coverslips were mounted with Fluoromount (Sigma) containing Hoechst 33342.

### 2.17 Cell apoptosis analysis

To induce cell apoptosis, indicated cells were incubated with Doxorubicin (Selleck) or glucose deprived (GD) media for 48 h. Cell apoptosis was detected using the Apoptosis Detection Kit (KeyGen Biotech, China) containing AlexFluor647 labelled Annexin V according to the manufacturer's instruction. Briefly, cells were harvested and rinsed with ice‐cold PBS, and resuspended in 200 μl of binding buffer. Ten microliters of AV‐ AlexFluor647 stock solution were added to cell suspensions and incubated for 30 min at 4°C. The cells were immediately analysed by a FACSCantoII flow cytometry (Becton–Dickinson, Mountain View, CA, USA).

### 2.18 ELISA

ELISA was performed to detect the cytokines in the culture medium. The kits for human IL6, 8 and TNFα detection were purchased from ExCell Biotech (China), and the assay was performed according to the manufacturer's instructions. The data were derived from 3 independent experiments with 4 biological replications. Absorbance was measured using a microplate reader (Thermo).

### 2.19 Luciferase assay

We used the reporter construct TOPflash, FOPflash (Upstate, Lake Placid, NY), NF‐κB RE, or TCF7L2 promoter luciferase reporters to evaluate β‐Catenin/TCF, NF‐κB transcriptional activity, or TCF7L2 promoter activity, respectively. Briefly, indicated cells were transiently transfected with the vectors. pRL‐TKRenilla luciferase plasmid (Promega) was also transfected as a constant control of total amount of transfected DNA in each well through all experiments. 48 h after transfection, cells were harvested and assayed for luciferase activity by the Dual Luciferase Reporter Assay System (Promega).

### 2.20 Tumour xenografts in nude mice

**FIGURE 1:**
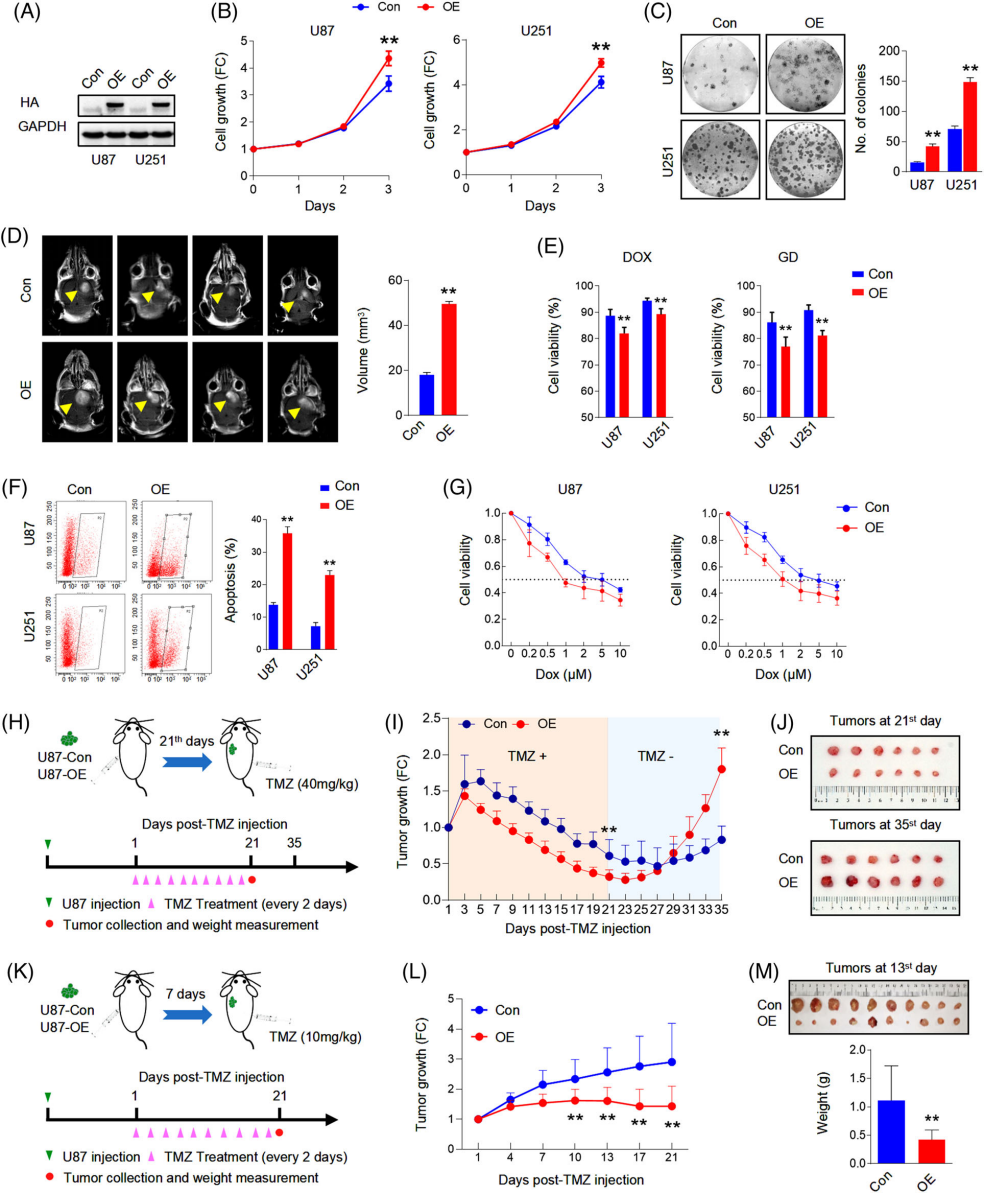
TCF4N promotes GBM cell tumourigenesis and chemosensitivity. (A) Western blot analysis of ectopic TCF4N expression in stable cell lines. GAPDH served as a loading control. (B) Cell growth assay of indicated stable cells using CCK‐8. (mean ± SD, *n* = 6, **p < 0.01). (C) Colony formation assay of indicated stable cells. (mean ± SD, *n* = 3, **p < 0.01). (D) Representative images of MRI of the orthotopic xenografts derived from the indicated cells. Yellow arrows indicate the tumours. The right panel shows the tumour volume analysis (mean ± SD, *n* = 4, **p < 0.01). (E) Cell viability assay of indicated cells treated with doxorubicin (Dox, 0.5 μM) or glucose deprivation (GD) for 48 h. Cell viability was evaluated by CCK‐8. (mean ± SD, *n* = 6, **p < 0.01). (F) Apoptosis assay of indicated cells treated with Dox (0.5 μM) for 48 h. Cell apoptosis was detected by flow cytometry (left panel). Statistical analysis was performed to calculate the apoptosis rate (right panel). (mean ± SD, *n* = 3, **p < 0.01). (G) Effect of TCF4N expression on Dox‐induced cell cytotoxicity. Indicated cell lines were incubated with various concentrations of Dox for 48 h and cell viability was evaluated by CCK‐8. (mean ± SD, *n* = 3). (H) Schematic illustration of the evaluation of in vivo tumour growth derived from the indicated cells responding to Temozolomide (TMZ) treatment. Mice were intracranially injected with U87 cells (either TCF4N overexpressing or control) and subsequently received 7 intraperitoneal injections of DMSO or TMZ (40 mg/kg) on the 21^st^ day post implantation. Tumour volume was monitored as indicated from the 21^st^ day. Tumours were collected on the 21^st^ day post TMZ‐treatment. (I) Growth curve of subcutaneous xenografts derived from the indicated cells. The tumour volume was measured at the indicated times after implantation. (mean ± SD, *n* = 7, **p < 0.01). (J) Representative images of subcutaneous xenografts collected on the 21^st^ and 35^th^ days after TMZ treatment. (K) Schematic illustration of the evaluation of tumour growth responding to TMZ treatment at the tumour‐initial stage. Mice were intracranially injected with the indicated cells and subsequently received 10 intraperitoneal injections of DMSO or TMZ (10 mg/kg) from the 7^th^ day post implantation. Tumour volume was monitored at the indicated times from the 7^th^ day. Tumours were collected on the 13^th^ day post treatment. (L) Growth curve of subcutaneous xenografts derived from the indicated cells. The tumour volume was measured at the indicated times after implantation. (mean ± SD, *n* = 10, **p < 0.01). (M) Representative images of subcutaneous xenografts collected on the 13^th^ day after TMZ treatment. The lower panel shows the tumour weight analysis (mean ± SD, *n* = 10, **p < 0.01).

Four weeks‐old female nude mice were obtained from the Shanghai Animal Center, Chinese Academy of Sciences and maintained under specific pathogen‐free conditions at The Affiliated Wuxi People's Hospital of Nanjing Medical University. To initiate tumours, 5 × 10^6^ cells in 100 μl of DMEM: Matrigel (8:1, v/v; BD Biosciences, Franklin Lakes, NJ) were injected s.c. into the flank of each nude mouse. For measuring the tumour growth responding to chemotherapy, the mice bearing xenografts were injected i.p. with Temozolomide (TMZ, Selleck) according to schematic illustration (Figure 1H,K, Figure 7J and Figure S7A). Tumour growth was monitored by measuring tumour diameters at the indicated times. At the final day of transplantation, mice were euthanised and tumours were collected, weighed and analysed. For intracranial brain tumour xenografts, dissociated GBM3 GSCs were stereotaxically implanted into the brains of individual mice. After 7 days of implantation, mice were randomly divided into 3 groups and received 10 i.p. injections of TMZ with or without 4N in the span of 15 days (Figure 7J). Twenty‐one days after implantation, tumours were monitored by magnetic resonance imaging (MRI, Siemens). For Kaplan–Meier analysis, mice were monitored daily and euthanised when the mice were moribund. Whole brains were removed, paraffin‐embedded, sectioned and then stained with hematoxylin and eosin (H&E). For measuring the effect of p65 or S536D on tumour growth, U87 cells stably expressing the firefly luciferase gene (5 × 10^5^ cells in 5 μl PBS) were stereotaxically implanted into the brains of individual mice. These cells expressed stable ectopic p65 or S536D. In additional experiments, to determine the effect of p65 or S536D on chemotherapy response, mice were injected i.p. once every 2 days with 40 mg/kg of TMZ from 3^rd^ day after implantation. Tumour growth was monitored via bioluminescent imaging using IVIS Spectrum system and quantified by Living Image Software. All animal care and handling procedures were performed in accordance with the National Institutes of Health's Guide for the Care and Use of Laboratory Animals and were approved by the Institutional Review Board of Nanjing Medical University. No mice were excluded from scoring. All animal experiments were conducted in a blinded manner.

### 2.21 Online cancer database analysis

The overview of *RELA* RNA expression in gliomas was based on the Rembrandt glioma dataset, TCGA RNA‐seq dataset and CGGA‐GBM RNAseq dataset. These datasets were downloaded from GlioVis data portal (http://gliovis.bioinfo.cnio.es/). Survival analysis targeting *RELA* expression in GBM was performed based on these datasets, and optimal cutoff value of *RELA* was produced by GlioVis. An additional GBM cohort expression and survival analysis targeting *RELA* expression were performed using the TCGA‐GBM database (Affymetrix Human Exon 1.0 ST) from Betastasis data portal (https://www.betastasis.com).

### 2.22 Statistical analysis

Data were expressed as mean ± SD. The difference between multiple groups was determined by a one‐way analysis of variance (ANOVA) followed by a Newman Keuls’ multiple comparison test. Survival analyses were performed using the Kaplan–Meier method with log‐rank test. All differences were considered significant when *p* < 0.05. SPSS 16.0 package (IBM) and GraphPad Prism 8.0 software (GraphPad Software) were used for all statistical analyses and data graphing, respectively.

## 3 RESULTS

### 3.1 TCF4N promotes GBM tumourigenesis, stem cell differentiation and chemosensitivity

To examine the roles of TCF4N in tumourigenesis, we generated an expression construct of TCF4N via chemical synthesis based on the sequence information from previous literature.^37^, ^38^ The reference sequence for TCF4N is shown in Data S2. We established TCF4N‐overexpressing stable cell lines from two GBM cell lines, U87 and U251, using the HA‐TCF4N lentivirus‐construct (Figure 1A). Contrary to expectations, the cell growth assay showed that ectopic TCF4N markedly promoted cell proliferation and colony forming in U87 and U251 cells (Figure 1B,C). The tumour promotion of TCF4N was further confirmed in the subcutaneous xenograft experiments. It demonstrated that the tumours derived from cells overexpressing TCF4N cells grew more quickly than those from control cells (Figure S1A,B). Accordingly, the orthotopic xenograft model confirmed the tumour promotion of TCF4N in vivo (Figure 1D). Considering the β‐catenin/TCF pathway is widely involved in the regulation of stem cell self‐renewal and differentiation,^39^ the functions of TCF4N in GBM stem cells were additionally examined. Here, we evaluated the effect of TCF4N on the initiation and self‐renewal of GBM cancer stem cells (CSCs) by continuously observing the growth process of 3D‐tumour spheroids (Figure S2A). GBM3, an established primary GBM cell line, as well as U251, can successfully form tumour spheres and continue to grow in the modified NSC medium containing EGF.^30^ Unexpectedly, we failed to observe a significant effect of TCF4N on the growth of primary tumour spheroids (GBM3p, U251p), suggesting no function of TCF4N on GBM CSC initiation (Figure S2A). To examine whether TCF4N functions on CSC self‐renew, the first passage of tumour spheroids was dissociated and re‐seeded to form the second passage of spheroids (GBM3s, U251s). As shown in Figure S2B, no significant difference between spheroids derived from cells with ectopic TCF4N and control cells was observed. As expected, TCF4N over‐expressed cells and control cells showed no significant difference in tumour sphere formation efficiency (Figure S2C). The double IF staining showed that spheroids derived from U251 expressed Nestin, a marker of neural stem cells and GFAP, a specific marker of astrocytes. However, spheroids from GBM3 expressed Nestin, but less GFAP (Figure S2D). Western blot analysis showed that ectopic TCF4N did not affect the expression of Nestin and GFAP in spheroids derived from these two cell lines (Figure S2E). Due to the low expression of GFAP, GBM3 CSCs were applied in the subsequent differentiation assay. GBM3 spheroids were detached on poly‐lysine coated coverslips and maintained in medium without EGF and bFGF to initiate differentiation. Double IF was performed using antibodies targeting three distinct neural cell markers, indicating that bFGF/EGF withdrawal induced a re‐expression of GFAP in GBM3 CSCs without the expression of neuronal or oligodendrocytic markers, indicating a spontaneous differentiation to astrocytes was initiated (Figure S2F). Further Western blot assay confirmed that TCF4N expression significantly promoted GFAP expression in GBM3 GSCs responding to bFGF/EGF withdrawal (Figure S2G). Next, we investigated whether TCF4N affects the cell response to DNA damage‐related stress. To this end, cell survival and apoptosis after chemotherapy or nutritional deprivation were examined. Although TMZ is a preferred alkylating agent for glioma/GBM, considering its antitumour effect depends on in vivo chemical conversion, we selected another widely used alkylating agent, Doxorubicin hydrochloride (Dox) as apoptosis was more consistently induced in the in vitro experiments. Cells were treated with a mild concentration of Dox (0.5 μM) or glucose deprivation (GD) for 48 h, and cell viability was measured. It showed that ectopic TCF4N increased cell loss upon Dox or GD treatment (Figure 1E). Flow cytometry analysis further showed that TCF4N introduction resulted in an increase in apoptosis after Dox treatment (Figure 1F). To confirm the contribution of TCF4N to the sensitivity of chemotherapy, cells were incubated with different concentrations (0–10 μM) of Dox. In both cell lines, ectopic TCF4N increased the cell loss in a Dox dose‐dependent manner (Figure 1G), confirming the chemosensitivity promotion of TCF4N in vitro. With this evidence, we next determined whether TCF4N contributes to chemotherapy sensitivity in vivo. Mice were subcutaneously injected with the indicated cells and tumours were allowed to develop and grow freely for 21 days. Next, mice received a course of intraperitoneal TMZ or DMSO at a regular dose (40 mg/kg) once every 2 days for 10 continuous injections (Figure 1H). tumour size was monitored daily from the 1^st^ injection of TMZ and was expressed as a relative volume. As shown in Figure 1I, tumours derived from both groups experienced a transient growth followed by a gradual decrease upon TMZ treatment. Corresponding to the in vitro apoptosis results, tumours derived from cells containing ectopic TCF4N resulted in much smaller tumours during TMZ treatment (Figure 1I,J), indicating that TCF4N increases the sensitivity to chemotherapy. Notably, there was a robust recurrence in both groups after TMZ withdrawal, and the recurrence of TCF4N expressing tumours was significantly faster than that of control tumours (Figure 1I). To better document TCF4N functions on chemotherapy sensitivity, we examined whether TCF4N affects chemosensitivity at early stages during tumour development. As shown in Figure 1K, mice were subcutaneously injected with the indicated cells and subsequently received a course of intraperitoneal TMZ or DMSO from the 7^th^ day after implantation, once every 2 days for 10 consecutive injections. Here, a low dose of TMZ (10 mg/kg) was used to investigate the tumour growth response to chemotherapy. By continuously tracking the tumour volume and analysing the tumour weight collected on the final day, we observed that the growth of tumours in the control group was not significantly suppressed by low‐dose of TMZ, while the growth of tumours in the TCF4N group was significantly slowed (Figure 1L,M), suggesting that TCF4N expression promotes tumour response to low‐dose of TMZ. Collectively, these results revealed that TCF4N plays a dual role as a tumour‐promotor and tumour suppressor under different conditions, this is particularly evident in TCF4N's role in conferring chemosensitivity.

### 3.2 TCF4N functions as a SASP inducer in GBM

**FIGURE 2:**
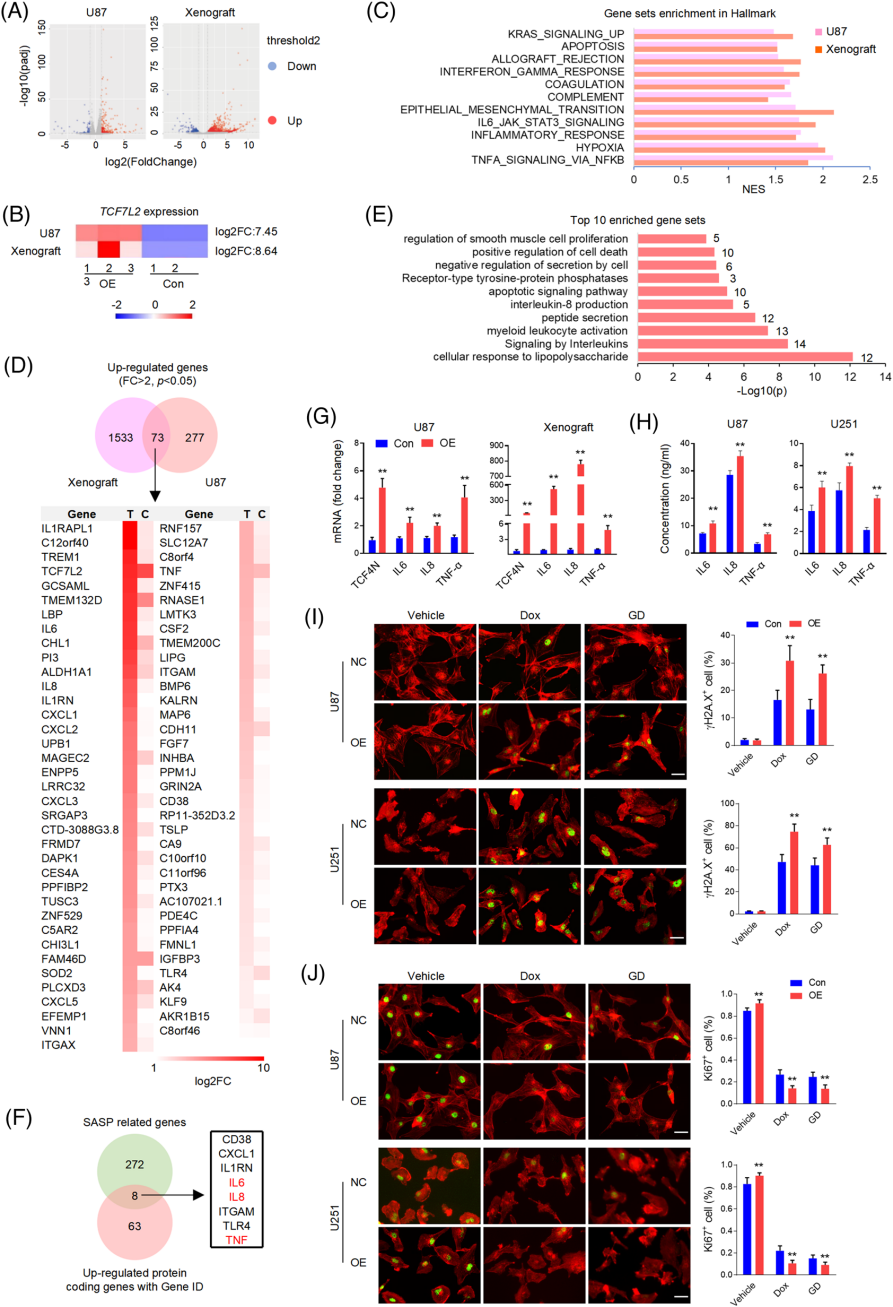
TCF4N promotes cell senescence associated cytokines expression. (A) The volcano plots of transcriptome sequencings show the differentially expressed genes in U87 cells and derived xenografts induced by ectopic TCF4N. (B) The heatmap derived from transcriptome sequencings show the fold change (FC) of *TCF7L2* in U87 cells and derived xenografts. (C) Gene set enrichment analysis (GSEA) shows the common enriched Hallmark sets between cells and tumours. (D) The 73 upregulated genes (FC > 2, *p* < 0.05) are listed in the volcano plot and heatmap. (E) Enrichment analysis provided the top 10 enriched gene sets based on the 73 common upregulated genes between tumours and cells. (F) Volcano plot showing the interactive analysis of the common upregulated protein coding genes and protein coding genes for SASP. (G) RT‐qPCR analysis showing the upregulation of the indicated cytokines both in cells and tumours expressing ectopic TCF4N. (mean ± SD, *n* = 3, **p < 0.01). (H) ELISA detection indicating the increase of cytokines production in cells expressing ectopic TCF4N. (mean ± SD, *n* = 4, **p < 0.01). (I) IF staining detecting the cellular senesce and DNA damage using phospho‐Histone H2A.X (γH2A.X). Cytoskeleton was labelled by F‐actin (Red). The indicated cells were treated with Dox (0.5 μM) or GD for 2 h. Cellular senesce was calculated by γH2A.X positive cell percentage. (mean ± SD, *n* = 6, **p < 0.01). Bars, 40 μM. (J) IF staining detected the cell proliferation using Ki67 antibody (Green). Cytoskeleton was labelled by F‐actin (Red). The indicated cells were treated with Dox (0.5 μM) or GD for 48 h. (mean ± SD, *n* = 6, **p < 0.01). Bars, 40 μM

We next explored the mechanism underlying the divergence of the oncogenic effects of TCF4N. Since TCF4N is defined as a β‐catenin binding protein,^37^, ^38^, ^40^ we asked whether TCF4N functions in GBM through mechanical interaction with β‐catenin. We first determined the interaction between TCF4N and β‐catenin. SW480 cells, a constitutively active β‐catenin containing cell line, were infected with HA‐TCF4N lentivirus. MDA‐MB‐453(MB453) cells, a human breast carcinoma cell line expressing ultralow levels of β‐catenin were used as a negative control. IP analysis revealed that TCF4N contained β‐catenin binding ability in SW480 cells (Figure S3A), while it failed to detect the significant interaction between TCF4N and β‐catenin in GBM cell lines (Figure S3B). Western blot analysis of cellular components indicated that the ectopic TCF4N was expressed both in the cytoplasm and nuclei of U87 and U251 cells, while β‐catenin was substantially localised in the cytoplasm (Figure S3C). The different distribution of these two proteins was further indicated by double IF (Figure S3D). Moreover, in both U87 and U251 cells, TCF4N introduction neither affected β‐catenin expression nor altered its activation (Figure S3E,F), suggesting that β‐catenin is not involved in the observed action of TCF4N in GBM cell lines. Our previous studies have disclosed that β‐catenin‐TCF4 interaction is not a dominant regulator of GBM, partly because of the non‐nuclear dominant expression of β‐catenin,^36^ implying a mechanism of TCF4N that functions in GBM in a β‐catenin‐independent manner. To uncover the potential mechanism of TCF4N in GBM, transcriptome sequencing was performed to compare the expression profiles of control cells and ectopic TCF4N‐expressing cells. To get more precise candidate molecules and mechanisms, the xenografts derived from control cells and ectopic TCF4N‐expressing cells were also evaluated via transcriptome sequencing. Volcano plots were generated to visualise the full list of genes analysed (Figure 2A). According to these two plots, significantly upregulated genes in TCF4N‐expressing cells and tumours were indicated by red dots, and down‐regulated genes were indicated by blue dots (Figure 2A). The heatmap showed the fold change (FC) of *TCF7L2*, partially indicating the overexpression of TCF4N (Figure 2B). Gene set enrichment analysis (GSEA) illustrated that the common enriched Hallmark sets between cells and tumours were associated with cytokine or chemokine‐related signalling pathways (such as NF‐κB and IL6/JAK/STAT3 pathway), apoptosis, and epithelial mesenchymal transition (Figure 2C). It also indicated that Wnt/β‐catenin signalling was only enriched in tumours (Figure S4), confirming that Wnt/β‐catenin regulation is not the dominant mechanism of TCF4N. Seventy three upregulated genes including *TCF7L2*(FC > 2, *p* < 0.05) were listed in the Venn diagram and heatmap (Figure 2D). Enrichment analysis disclosed that these genes were associated with interleukin production and downstream pathways, apoptosis and cell death pathways, receptor tyrosine‐protein phosphatases, etc. (Figure 2E). According to GeneCards (https://www.genecards.org/Search/Keyword?queryString = SASP&pageSize = ‐1&startPage = 0), 8 of the 71 upregulated protein coding genes (excluding *TCF7L2*) were related to the SASP (Figure 2F). Among the SASP associated genes, the major components such as IL6, IL8 and TNF were subjected to validation by mRNA expression (Figure 2G) and protein secretion (Figure 2H). In addition to these 8 proteins, CXCL2, CXCL3, CXCL5 and IGFBP3 were also major components of SASP.^41^, ^42^ SASP is a typical feature of chronic inflammation induced by tumour cellular senescence. Accumulating evidence shows that senescent cells have dual effects on cancer after acquiring SASP.^43^, ^44^ On this premise, we speculated that TCF4N regulates SASP in GBM cells. To determine whether SASP was derived from increased cellular senescence induced by TCF4N, phospho‐Histone H2A.X (Ser139) antibody was used to label the senescent cells. As shown in Figure 2I, no comparable difference in H2A.X positive cell numbers was found between control cells and ectopic TCF4N expressing cells, suggesting that TCF4N does not induce cellular senescence under normal conditions. While under Dox or GD, cells containing ectopic TCF4N had higher phospho‐Histone H2A.X expression, suggesting that TCF4N accelerates DNA damage under stress. Since proliferation and senescence are two opposing processes, IF staining of Ki67 was performed to measure the proliferation upon TCF4N introduction under various conditions (Figure 2J). The results showed that TCF4N expression resulted in an enhancement of cell proliferation under stress‐free conditions. This result, in combination with the H2A.X data indicated that TCF4N induced SASP is not associated with cellular senescence under normal conditions. Correlated with H2A.X expression, Dox or GD treatment induced a significant decline in cell proliferation in control cells, a decline that was further increased in TCF4N overexpressing cells. These data suggest that TCF4N functions as a SASP inducer, resulting in the promotion of GBM cell proliferation and apoptosis under stress‐free and challenging conditions, respectively.

### 3.3 TCF4N promotes NF‐κB activation by regulating p65 S536 phosphorylation, nuclear translocation and stability

**FIGURE 3:**
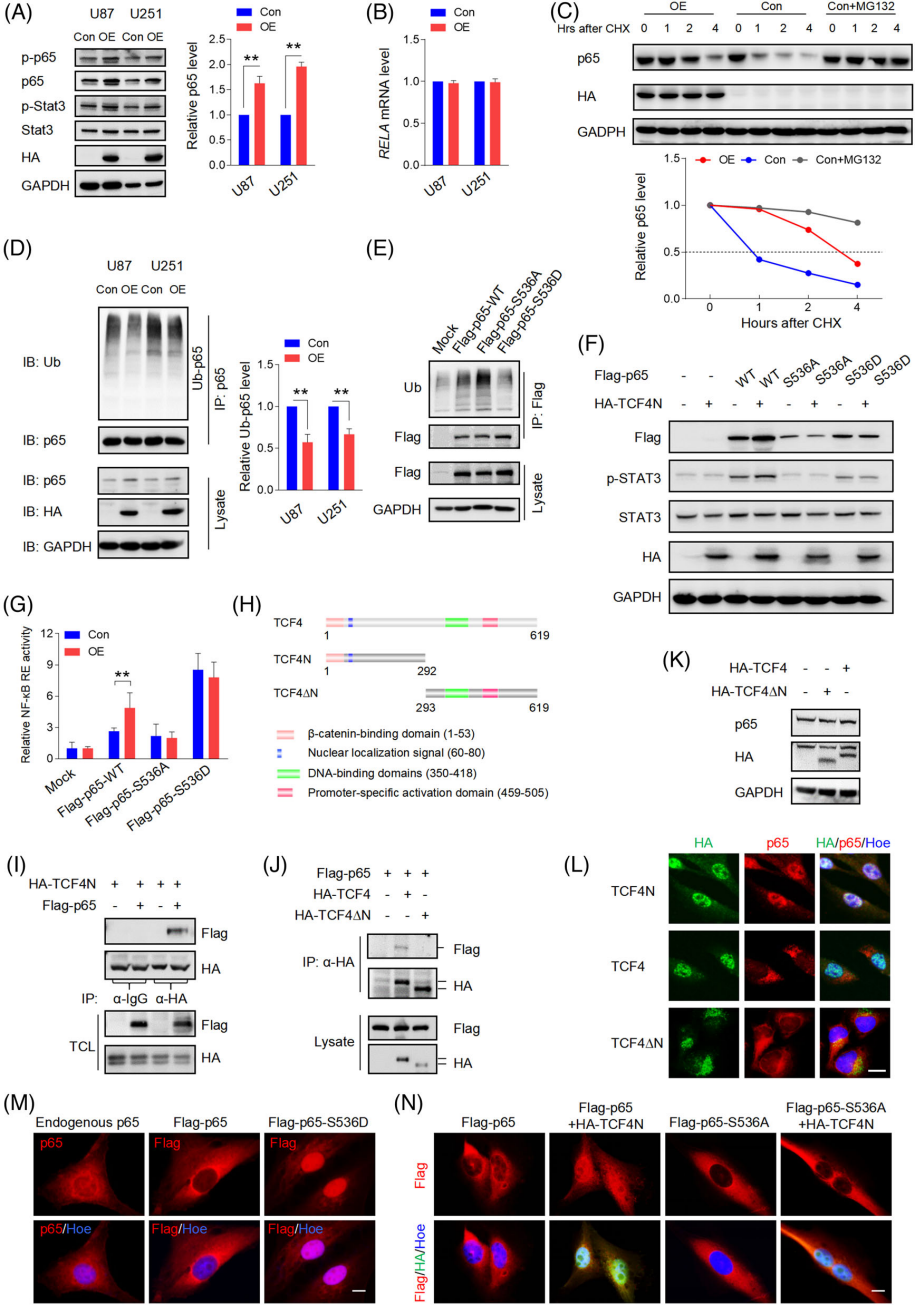
TCF4N promotes NF‐κB activation by regulating p65 S536 phosphorylation, nuclear translocation and stability. (A) Western blot analysis indicating the activation of p65 and Stat3 in U87 and U251 cells expressing ectopic TCF4N. GAPDH served as a loading control. The right panel shows the statistical result of p65 protein level. *n* = 3, **p < 0.01. (B) RT‐qPCR analysis showing ectopic TCF4N had no effect on *RELA* (p65) mRNA expression. (C) Western blot analysis of p65 expression in U87 cells with ectopic TCF4N. Cells were treated with cycloheximide (CHX, 100 μg/ml) with or without MG132 (20 μM) for the indicated hours before harvest (left panel). The alteration of relative p65 protein level was shown in the right panel. (D) Ubiquitination analysis of p65. The indicated cells were incubated with MG132 (20 μM) for 6 h before harvest. Ubiquitin (Ub)‐binding p65 was immunoprecipitated (IP) by p65 antibody and detected by Ub antibody. Target proteins in the total lysate were also detected by the indicated antibodies. (mean ± SD, *n* = 3, **p < 0.01). (E) Ubiquitination analysis of indicated ectopic p65 in p65‐KO cells. Ub‐binding p65 was immunoprecipitated by Flag antibody and detected by Ub antibody. Target proteins in the total lysate were also detected by the indicated antibodies. (F) Western blot analysis of indicated proteins in p65‐KO cells expressing ectopic TCF4N. GAPDH served as a loading control. (G) Transcriptional activity analysis of NFκB using luciferase reporter system. *n* = 3, **p < 0.01. (H) Diagram of indicated TCF4 constructs. (I) IP analysis indicating TCF4N binding of p65. U87 cells expressing TCF4N were transfected with an empty vector or a p65 vector (Flag‐p65) and followed with an IP analysis using HA antibody. IgG antibody was used as a negative control. (J) Analysis of the interaction between the indicated constructs and p65. U87 cells co‐expressing of p65 and TCF4 or TCF4∆N vectors and were subjected to IP analysis using HA antibody. (K) Western blot analysis of p65 level in U87 cells expressing ectopic TCF4 or TCF4∆N. (L) Double IF staining showing the distribution of p65 in U87 cells upon expressing the indicated TCF4 constructs. Bars, 20 μM. (M) IF staining showing the cellular localization of endogenous or ectopic p65 in U87 cells. Scale bars, 10 μm. (N) IF staining showing the cellular expression of ectopic p65 in p65‐KO cells with or without TCF4N expression. Scale bars, 10 μm

NF‐κB and Stat3 are key signalling molecules mediating cellular senescence and SASP.^45^ Additionally, they play important roles in promoting tumourigenesis and development, as well as drug resistance.^6^, ^46^ The finding that TCF4N overexpression promotes typical SASP chemokines led to investigating whether TCF4N links the activation of NF‐κB and Stat3. As shown in Figure 3A, ectopic TCF4N expression induced an increase of Stat3(Tyr705) and p65(Ser536) phosphorylation. Notably, we observed an upregulation of total p65 protein induced by TCF4N expression, but no change in total Stat3 expression. NF‐κB signalling plays important roles in triggering SASP,^47^ prompting us to explore the mechanism involved in TCF4N regulation of p65 expression. RT‐qPCR results refuted that TCF4N regulates the expression of p65(*RELA*) at a transcriptional level (Figure 3B). Given that p65 is regulated by the ubiquitin‐proteasome pathway,^48^ we speculated that the impairment of proteasomal degradation may be the underlying mechanism of TCF4N‐mediated p65 upregulation. To this end, we examined the decay rate of p65 using cycloheximide (CHX) to inhibit protein synthesis. After treatment with or without the proteasome inhibitor MG132 for 4 h, U87 cells were treated with CHX for the indicated time (Figure 3C). Results indicated that p65 was degraded rapidly, with more than 50% becoming degraded after 1 h. This degradation was greatly reduced in the presence of MG132, indicating a proteasomal degradation pathway was the major mechanism affecting the stability of p65. It also showed that ectopic TCF4N extended the half‐life of p65. Furthermore, the ubiquitination assay demonstrated that p65 was modified by ubiquitination in U87 and U251 cells, and that p65 ubiquitination and degradation were inhibited by the introduction of TCF4N (Figure 3D). Next, we asked whether TCF4N‐protected p65 is related to the increase of S536 phosphorylation of p65. To this end, we established CRISPR/Cas9 mediated p65 knock‐out (KO) U87 cell lines (Figure S5A), and the sg3 cell line was applied in the subsequent exploration. The cells were re‐expressed with wide‐type (Flag‐p65‐WT) p65, S536 phosphomimetic mutation (Flag‐p65‐S536D), or the non‐phosphomimetic mutation (Flag‐p65‐S536A) containing a synonymous mutation targeting the sg3 sequence. The following ubiquitin assay showed that p65‐S536A possessed higher ubiquitination, and p65‐S536D has the lowest ubiquitination (Figure 3E). TCF4N introduction failed to increase the expression of p65 in cells containing p65‐S536A, as well as in those containing p65‐S536D (Figure 3F). The results also showed that p65‐S536D markedly promoted the phosphorylation of Stat3, while the phosphorylation of Stat3 in cells with p65‐S536A was even lower than it in cells expressing WT. This low Stat3 phosphorylation was not improved by the intervention of TCF4N (Figure 3F). As a result, NF‐κB transcriptional activity was significantly increased in p65‐S536D expressing cells, but not in cells expressing p65‐S536A (Figure 3G). TCF4N expression failed to affect the NF‐κB transcriptional activity in cells containing p65‐S536D or p65‐S536A. Next, we asked whether there was an interaction between TCF4N and p65. We additionally constructed a TCF4 vector and a TCF4∆N vector expressing a truncated mutant without the TCF4N coding sequence (Figure 3H). As shown in Figure 3I, IP analysis indicated that TCF4N can bind p65. Interestingly, a weak interaction between TCF4 and p65 was also observed while no detectable interaction was found between TCF4∆N and p65(Figure 3J). Western blot results showed that TCF4 or TCF4∆N failed to affect p65 expression in U87 cells (Figure 3K), indicating that TCF4N‐induced p65 protein elevation is its unique function. Moreover, double IF staining disclosed that TCF4N induced a nuclear‐translocation of p65 and that the interaction of TCF4N and p65 happened in the nuclei of U87 cells (Figure 3L). Although ectopic TCF4 was expressed in nuclei, it failed to induce p65 nuclear‐translocation. Due to lacking the nuclear localization signal (NLS), TCF4∆N was undoubtedly distributed in the cytoplasm and did not alter the distribution of p65. Next, we asked whether TCF4N induces p65 nuclear‐translocation and stability through the regulation of S536 phosphorylation. In U87 cells, endogenous p65 was mostly localised in the cytoplasm, and this distribution was also reflected in cells with ectopic p65. Meanwhile, p65‐S536D showed a predominately nuclear expression in U87 cells (Figure 3M). In re‐expressed p65 cells, it showed that both p65‐WT and p65‐S536A maintained the dominant cytoplasmic localization (Figure 3N). TCF4N introduction predictably increased the nuclear transport of WT, while failing to induce p65‐S536A nuclear translocation, suggesting that S536 is a vital site for TCF4N action on p65 nuclear translocation. Considering the phosphorylation of S536 promotes p65 nuclear translocation, elevated transcriptional activity and induction of apoptosis and senescence,^49^ these data suggest that TCF4N promotes NF‐κB activity, SASP and Stat3 activation by inducing p65 phosphorylation at S536 and subsequent nuclear translocation.

### 3.4 S536 phosphorylation of p65 is essential for the dual functions of TCF4N in GBM

**FIGURE 4:**
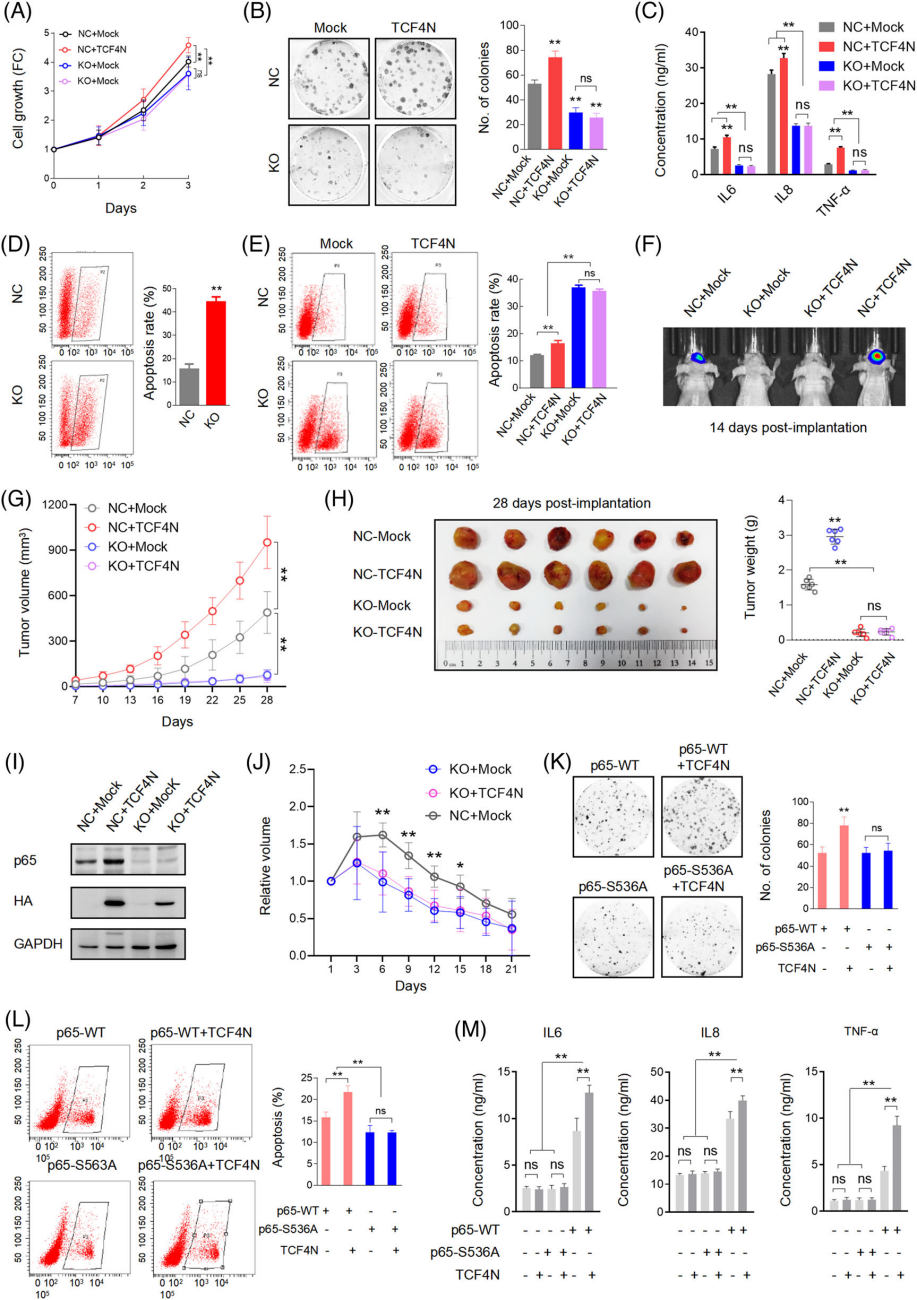
S536 phosphorylation of p65 is essential for the dual roles of TCF4N in GBM. (A) Cell growth assay showing TCF4N introduction fails to increase the proliferation inhibition of U87 cells induced by p65 knock‐out. NC, negative control; KO, p65 knock‐out U87 cells. (mean ± SD, *n* = 3, **p < 0.01; ns, no significance). (B) Colony formation assay of the indicated cells. (mean ± SD, *n* = 3, **p < 0.01; ns, no significance). (C) ELISA detection indicating TCF4N introduction fails to rescue the decline of cytokine production leading to p65 depletion. (mean ± SD, *n* = 6, **p < 0.01). (D) Apoptosis assay of p65 KO cells and NC cells. (mean ± SD, *n* = 3, **p < 0.01). (E) Apoptosis assay of the indicated cells treated with Dox (0.5 μM) for 48 h. (mean ± SD, *n* = 3, **p < 0.01). (F) Bioluminescent images of intracranial xenografts derived from the implantation of the indicated cells. (G) Growth curve of subcutaneous xenografts derived from the indicate cells. The tumour volume was measured at the indicated times after implantation. (mean ± SD, *n* = 6, **p < 0.01). (H) Representative images of subcutaneous xenografts collected on the 28^th^ day after implantation. The right panel shows the tumour weight analysis (mean ± SD, *n* = 6, **p < 0.01). (I) Western blot analysis of p65 and TCF4N expression in the subcutaneous xenografts. (J) Growth curve of subcutaneous xenografts derived from the indicate cells. The tumour volume was measured at the indicated times after TMZ injection. The subcutaneous xenografts derived from the indicated cells were allowed to grow for 28 days and subsequently received a course of TMZ (40 mg/kg) once every 2 days for 10 continuous injections. (mean ± SD, *n* = 10, *p < 0.01, **p < 0.01). (K) Colony formation assay of the cells expressing indicated constructs. (mean ± SD, *n* = 3, **p < 0.01; ns, no significance). (L) Apoptosis assay of the indicated cells treated with Dox (0.5 μM) for 48 h. (mean ± SD, *n* = 3, **p < 0.01; ns, no significance). (M) ELISA detection indicating the cytokine production in indicated cells (mean ± SD, *n* = 6, **p < 0.01)

Although widely connected to tumourigenesis and chemotherapy resistance, many studies have demonstrated that NF‐κB can inhibit tumour growth, promote apoptosis and chemosensitivity.^6^, ^7^, ^10^, ^50^ The current finding that TCF4N can increase p65 expression and phosphorylation postulates that p65 is a central component in mediating TCF4N dual functions in GBM. To explore this hypothesis, we determined whether TCF4N is still functional in p65 KO cells or not. The three established U87 p65 KO cell lines showed a significant decrease in capacity in colony formation compared with negative control cells (Figure S5B). Since these cells showed similar colony formation capacity, the mixed cells containing the same proportions were used in the following experiments. As shown in Figure 4A,B, p65 deletion significantly inhibited cell growth and colony formation. Although promoting negative control cell (NC) proliferation and colony growth, ectopic TCF4N failed to reverse the growth inhibition induced by p65 depletion. Accordingly, p65 KO resulted in a significant decrease in SASP chemokine secretion and TCF4N failed to rescue the reduction of chemokine secretion in p65 KO cells (Figure 4C). Next, we asked whether TCF4N promoting GBM chemotherapy sensitivity is dependent on p65. Under stress‐free conditions, we found that p65‐depleted U87 cells exhibit a higher apoptosis rate than negative control cells (Figure 4D). In terms of in vitro chemosensitivity, the apoptosis induced by Dox in p65 KO cells was further amplified (Figure 4E), suggesting that p65 is key factor for cell survival. Interestingly, TCF4N introduction failed to increase the apoptosis of p65‐depleted cells in response to Dox, providing a clue that the increased apoptosis of TCF4N depends on p65. Subsequently, the in vivo tumour model was applied to determine the effects of p65 on tumourigenesis and development. The intracranial xenograft model showed that at the 14^th^ day post‐implantation, no obvious tumour could be detected in mice implanted with p65‐depleted cells, and ectopic TCF4N expression failed to rescue this inhibition (Figure 4F). Considering the intracranial tumourigenesis of p65‐depleted cells was too weak to observe the potential tumour‐promoting effect of TCF4N, the subcutaneous xenograft model was adopted for further observation (Figure 4G,H). It showed that p65‐depleted cells derived tumours grew more slowly than negative control cells derived tumours (Figure 4G). Tumour collection on the final experimental day indicated that p65‐depleted cells formed much smaller tumours than control cells (Figure 4H), suggesting p65 is dispensable for GBM tumourigenesis and development. Ectopic TCF4N expression resulted in the promotion of tumour growth of negative control cells while failing to promote the growth of tumours derived from p65‐depleted cells. Accordingly, ectopic TCF4N expression resulted in an elevation of p65 expression in tumours from negative control cells, while failing to rescue p65 expression in tumours derived from p65‐depleted cells (Figure 4I). These data either demonstrate that p65 or NF‐κB is essential for tumourigenesis and progression^51^, ^52^ or reveal that tumour promotion of TCF4N is p65‐dependent. To further confirm that the chemosensitivity promotion of TCF4N is dependent on p65, the in vivo chemotherapy response was examined in the subcutaneous xenografts derived from p65 KO cells as shown in Figure 4G. The tumours were allowed to grow for 28 days and subsequently received a course of intraperitoneal TMZ at regular dose (40 mg/kg), once every 2 days for 10 continuous injections. As indicated in Figure 4J, tumours derived from p65 KO cells showed a tendency to be more sensitive than those derived from control cells (NC + Mock). On the other hand, we failed to observe a significant difference between tumours derived from KO + Mock and KO + TCF4N expressing cells, indicating that depletion of p65 blocks the chemosensitivity promotion of TCF4N. Next, we asked whether TCF4N actions in GBM is depended on S536 phosphorylation of p65. To this end, U87 KO cells were re‐expressed p65‐WT or p65‐S536A construct with or without TCF4N. Colony growth assay indicated that S536A expression had no effect on the colony growth capacity, even in cells with ectopic TCF4N (Figure 4K). Interestingly, Dox‐induced apoptosis in cells expressing p65‐S536A was lower than it in p65‐WT expressing cells (Figure 4L). TCF4N intensified the Dox‐induced apoptosis of cells expressing p65‐WT but failed to affect the apoptosis of cells with p65‐S536A. Accordingly, p65‐S536A re‐expression did not elevate SASP chemokines production, and TCF4N expression did not increase the production of SASP chemokines from cells with p65‐S536A (Figure 4 M). Collectively, these results demonstrate that S536 phosphorylation of p65 is essential for TCF4N functioning in GBM.

### 3.5 S536 phosphorylation drives p65 nuclear localization and endows dual functions in tumourigenesis and chemosensitivity

**FIGURE 5:**
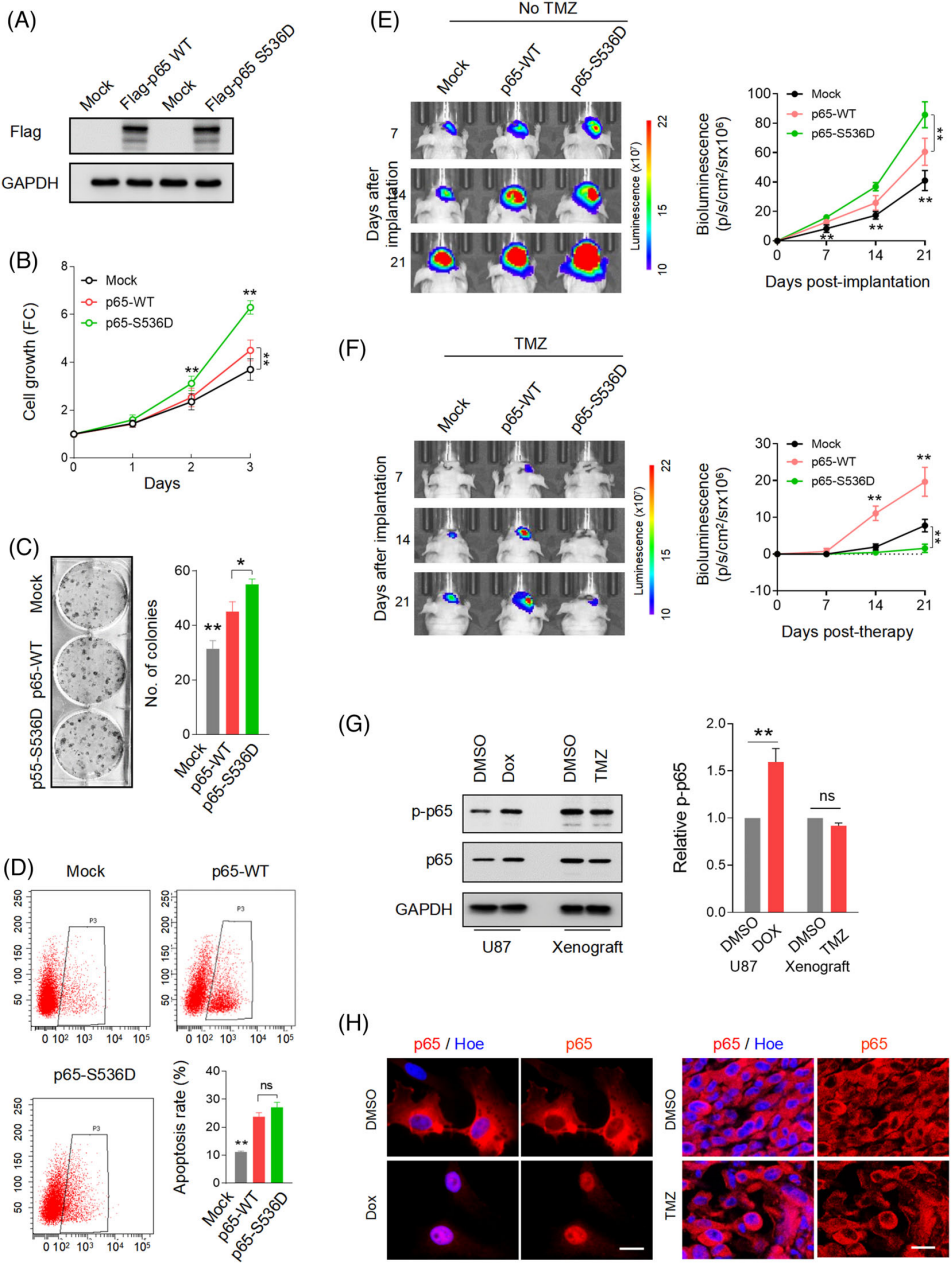
S536 phosphorylation is required for p65 nuclear translocation and dual functions of NF‐κB in GBM. (A) Western blot analysis of ectopic Flag‐p65‐WT or Flag‐p65‐S536D expression in U87 cells. (B) Cell growth assay showing p65‐WT or p65‐S536D overexpression promotes U87 cell proliferation. (mean ± SD, *n* = 3, **p < 0.01). (C) Colony formation assay of the indicated cells. (mean ± SD, *n* = 3, *p < 0.05, **p < 0.01). (D) Apoptosis assay of the indicated cells treated with Dox (0.5 μM) for 48 h. (mean ± SD, *n* = 3, **p < 0.01; ns, no significance). (E) Bioluminescent images of intracranial xenografts derived from the implantation of the indicated cells (left panel) and quantification of tumour volume (right panel). (mean ± SD, *n* = 6, **p < 0.01). (F) Intracranial tumourigenesis model showing the tumours derived from the indicated cells responding to TMZ treatment. TMZ (40 mg/kg) treatment was given once every 2 days from the 3^rd^ day after cell implantation. (mean ± SD, *n* = 6, **p < 0.01). (G) Western blot analysis of p65 phosphorylation (S536) in U87 cells and tumours using the indicated antibodies. GAPDH served as a loading control. U87 cells were treated with Dox (0.5 μM) for 6 h. The tumours were derived from mice with subcutaneous xenografts of U87 and treated with 5 consecutive TMZ (40 mg/kg) injections. The right panel shows the quantitative results of p65 phosphorylation. (mean ± SD, *n* = 3, **p < 0.01; ns, no significance). (H) IF staining of p65 in U87 cells (left) or derived xenografts (right) with or without treatment. Bars, 20 μm

Since p65 S536 phosphorylation is essential for TCF4N actions in GBM, the key to elucidating the mechanism of TCF4N's dual regulation is to decipher whether S536 phosphorylation of p65 has both cancer‐promoting and chemo‐sensitizing effects. To this end, U87 cells overexpressing p65‐WT or p65‐S536D were used for the subsequent functional studies (Figure 5A). Cell growth assay showed that p65‐WT overexpression resulted in an elevation of cell proliferation and p65‐S536D expression induced a much stronger proliferation ability (Figure 5B), as also reflected in colony formation capacity (Figure 5C). However, the apoptosis assay indicated that both p65‐WT and p65‐S536D expression resulted in an increased cell apoptosis induced by Dox treatment, while no difference was observed between cells expressing p65‐WT and p65‐S536D (Figure 5D). Next, the intracranial tumour model was used to confirm the dual function of p65‐WT as well as p65‐S536D. The tumourigenesis model indicated that p65‐WT overexpression significantly promoted tumour growth, and that p65‐S536D introduction further strengthened the tumour‐promotion of p65‐WT (Figure 5E). To explore the effect of p65‐WT and p65‐S536D on chemotherapy sensitivity, TMZ was given from the 3^rd^ day after intracranial injection and tumour growth was continuously monitored. Unlike the results observed in apoptosis experiments in vitro, it showed that the tumours overexpressing p65‐WT had a lower therapeutic response to TMZ compared to those derived from Mock cells (Figure 5F). More importantly, the growth of tumours expressing p65‐S536D was significantly inhibited compared with the tumours derived from Mock group, indicating that p65‐S536D significantly enhanced the reactivity to TMZ (Figure 5F). In view of the inconsistent response of p65 overexpressed cells to chemotherapy in vitro and in vivo, we asked whether it resulted from different response of phosphorylation to treatment. As shown in Figure 5G, Dox treatment drove a significant increase of p65 phosphorylation at S536 in U87 cells, while TMZ treatment failed to alter the phosphorylation of p65 at S536, as well as the total level of p65 in U87‐derived tumours. Corresponding to the different effects on S536 phosphorylation, Dox treatment led p65 expression from the cytoplasm to the nucleus in cultured U87 cells, while the addition of TMZ did not alter the cytoplasmic expression of p65 in tumours derived from U87 cells (Figure 5H). Moreover, in intact GBM cells and p65‐WT overexpressing cells, p65 was mainly expressed in the cytoplasm (Figure 3 M), revealing that p65 is not activated in the absence of stimulation by default. Meanwhile, p65‐S536D was predominately expressed in nuclei. Collectively, these results demonstrate that S536 phosphorylation endows p65 dual effects in promoting tumourigenesis and chemosensitivity in GBM.

### 3.6 Nuclear p65 promotes TCF4N expression and acts as a promising predictor for GBM

**FIGURE 6:**
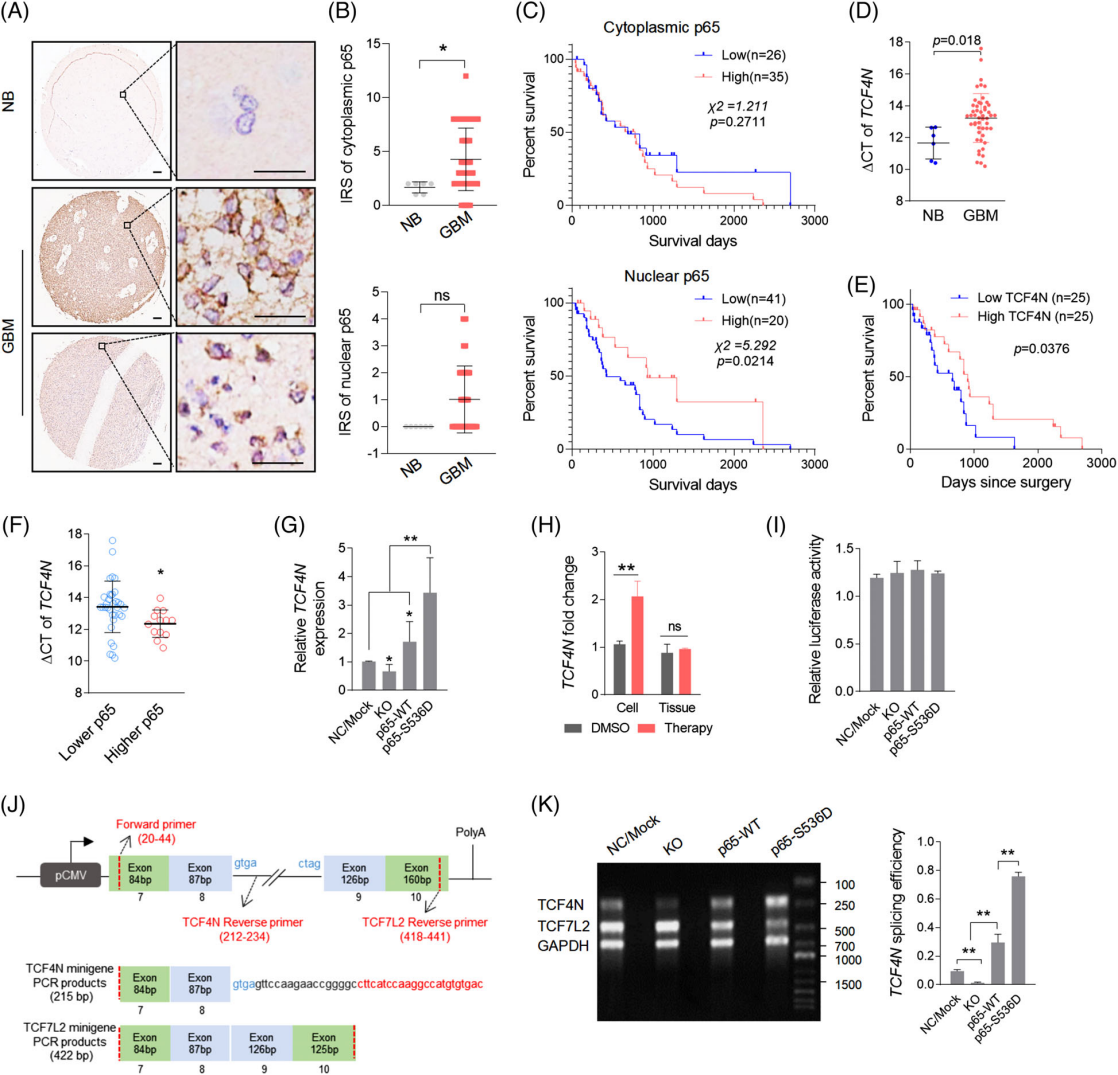
Nuclear p65 promotes TCF4N expression and works as a promising predictor for GBM. (A) Representative images of p65 immunohistochemistry (IHC) in normal brain and GBM tissues. Scale bars, 100 μm. (B) IHC analysis of cytoplasmic (upper panel) or nuclear‐p65 (lower panel) in GBM (*p < 0.05, Unpaired *t*‐test; ns, no significance). (C) Overall survival analysis based on cytoplasmic (upper panel) or nuclear‐p65 (lower panel) expression levels in GBM tissues. Groups were ranked according to the IHC scores. The cutoff was set at IRS 4 or 1 separately (Log‐rank). (D) RT‐qPCR analysis of TCF4N expression in normal brains and GBM tissues. (E) Overall survival analysis based on TCF4N expression levels in GBM tissues (Log‐rank).(F) Analysis of *TCF4N* mRNA expression based on nuclear‐p65 expression levels in GBM tissues (*p* = 0.0306). (G) RT‐qPCR analysis of *TCF4N* expression in U87 cells expressing the indicated constructs (mean ± SD, *n* = 3, *p < 0.05, **p < 0.01). (H) RT‐qPCR analysis of TCF4N expression in U87 cells and derived xenografts with the indicated treatment (mean ± SD, *n* = 3, **p < 0.01). (I) Luciferase assay of *TCF7L2* promoter activity in indicated cells. (J) The TCF7L2 minigene and primer designs. The construct was linked to a CMV promoter and contained four exons. The splicing product was detected by PCR using common forward primer and indicated reverse primer. (K) RT‐PCR analysis of transcripts in indicated cells (left) and the quantitative analysis of *TCF4N* splicing efficiency as calculating the expression of *TCF4N* to *TCF7L2* (right; mean ± SD, *n* = 3, **p < 0.01)

S536 phosphorylation driven p65 nuclear translocation suggests that nuclear p65 is involved in the enhancement of TMZ therapy sensitivity. High nuclear or cytoplasmic expression of p65 predicts better or worse survival in cancers.^53^, ^54^ These backgrounds suggest that patients with higher nuclear p65 may reveal those who may demonstrate a more promising chemotherapy response. To determine this possibility, the clinical significance of p65 in GBM was examined. The significant *RELA* transcriptional expression was characterised in different glioma datasets. *RELA* is highly expressed in gliomas covering various datasets, including different histologies (Figure S6A) and GBM (Figure S6B,C). Survival analysis revealed that *RELA* fails to connect survival prediction of GBM patients (Figure S6D–F). In a TCGA‐GBM dataset (Affymetrix Human Exon 1.0 ST) derived from Betastasis (https://betastasis.com/glioma/tcga_gbm/gene_survival_association_affymetrix_human_exon_10_st/), *RELA* was detected to be upregulated in GBM according to all subtypes of GBM (Figure S6G), while higher *RELA* predicts better survival in GBM patients (Figure S6H). One notable result was that in GBM patients receiving chemotherapy, the survival outcome of patients with higher *RELA* is significantly better than that of patients with lower *RELA* (Figure S6I). To further determine the clinical relevance, we explored the expression of *RELA*/p65 in primary GBM tissues. Immunohistochemical (IHC) results of gliomas derived from the Human Protein Atlas showed that p65 was expressed in the cytoplasm or membrane (https://www.proteinatlas.org/ENSG00000173039‐RELA/pathology/glioma#Location). The primary GBM tissue microarray was used to examine the p65 protein expression (Figure 6A). It showed that p65 was mainly expressed in the cytoplasm of tumour cells, and scattered in the nuclei, consistent with the online immunohistochemical data from the Human Protein Atlas. The cytoplasmic expression of p65 in GBM was significantly higher than it in normal brain tissues, while it was not correlated to the survival of GBM patients. For nuclear p65, although there was no significant difference, it was still observed that the survival outcome of patients with higher nuclear‐p65 connected to better survival outcomes (Figure 6B,C).We then examined the relevance of TCF4N and nuclear p65 based on the IHC of p65 and qPCR results of *TCF4N*. Nucleotide sequence alignment of *TCF4N* and *TCF7L2* isoform v1 indicated that *TCF4N* has a divergent sequence at the C‐terminal containing a terminator (Figure S7A). According to the unique C‐terminal sequence of *TCF4N*, the specific primers crossing the termination codon were designed for detecting *TCF4N* expression via qPCR (Figure S7B). The qPCR result showed that *TCF4N* was significantly downregulated in GBM compared with it in normal brains (Figure 6D). Survival analysis performed on 50 patient samples with detailed survival information (Data S1 and Table S1) indicated that lower *TCF4N* predicted unfavourable survival outcome regardless of age and sex (Figure 6E; log‐rank survival analysis; *p* = 0.0376). Moreover, *TCF4N* was upregulated in cases with higher nuclear‐p65 expression (Figure 6F), suggesting that p65 potentially connects to the expression of TCF4N in GBM. Interestingly, TCF4N was significantly increased in cells expressing p65‐S536D, and p65 depletion (KO) or overexpression (p65‐WT), resulting in a moderate decline or upregulation of TCF4N in U87 cells (Figure 6G). Additionally, TCF4N was found to be significantly upregulated in cells treated with Dox but not in TMZ treated xenografts (Figure 6H), confirming that p65 S536 phosphorylation promotes TCF4N expression. Given that the *TCF7L2* isoform is derived from alternative splicing of pre‐mRNA,^38^, ^40^ the finding that p65 regulates TCF4N expression led to determine whether nuclear p65 promotes alternative splicing of RNA towards *TCF4N*. Since transcripts of *TCF7L2* share the same promoter, we constructed a *TCF7L2* promoter reporter vector containing a firefly luciferase gene under the control of *TCF7L2* promoter. As shown in Figure 6I, the result of promoter reporter assay excluded the possibility that p65 regulates the transcription of *TCF7L2*. To examine the involvement of p65 in regulating *TCF4N* alternative splicing, the minigene system was produced as shown in the illustrator (Figure 6J), and the splicing was monitored by PCR products using indicated primers (Figure 6K). By calculating the splicing ratio of *TCF4N* to *TCF7L2*, it disclosed that p65 positively regulates the alternative splicing of *TCF4N* (Figure 6K). Therefore, we disclosed a novel regulatory loop between TCF4N and p65 which promotes GBM tumourigenesis and chemosensitivity by impacting p65 S536 phosphorylation, nuclear‐translocation and the dominant splicing of *TCF4N*.

### 3.7 Eukaryotic recombinant cell‐penetrating protein possesses functions as endogenous TCF4N

**FIGURE 7:**
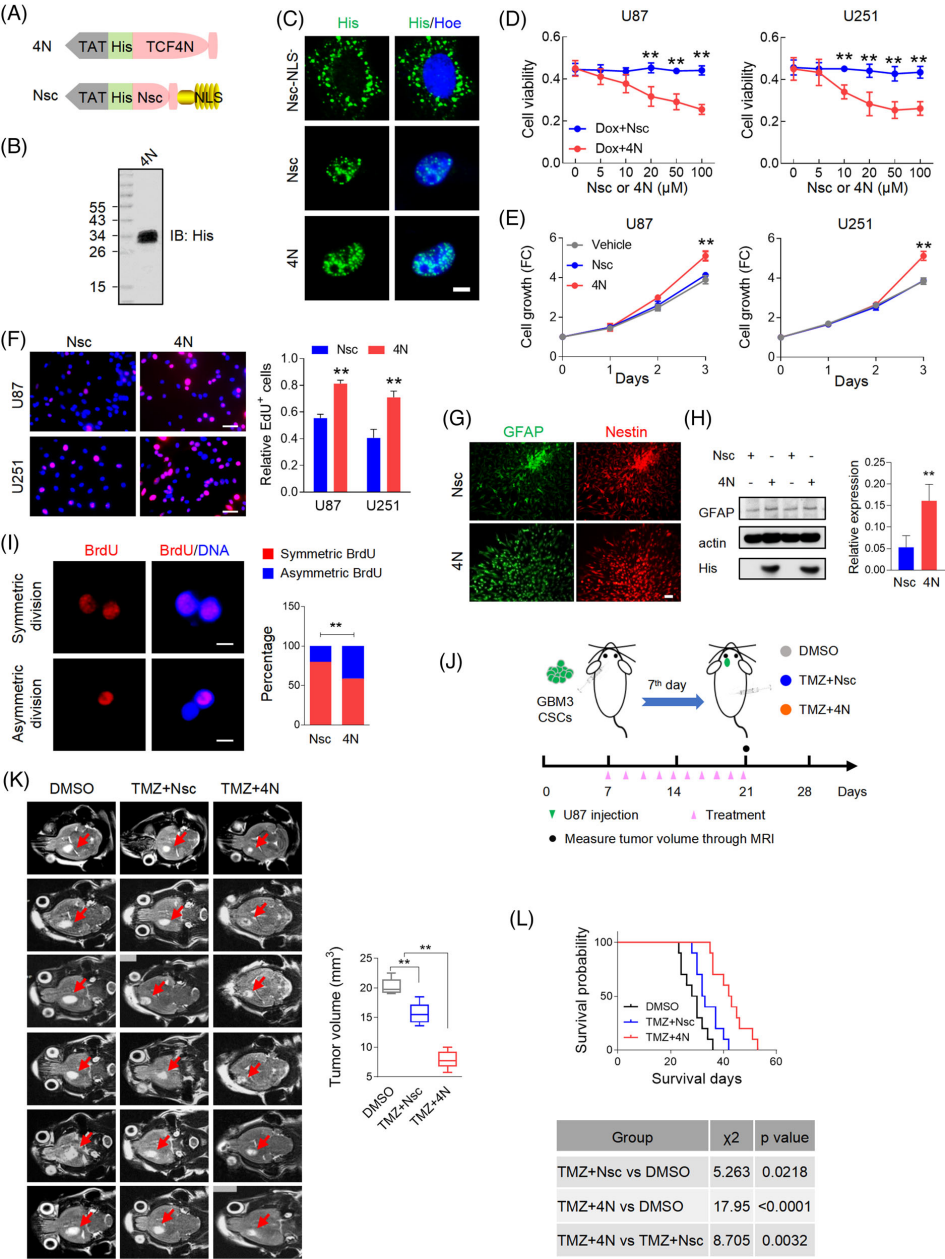
Eukaryotic recombinant cell‐penetrating TCF4N increases chemosensitivity of GBM stem cells in vitro and in vivo. (A) The eukaryotic recombinant cell‐penetrating TCF4N (4N) and its negative control (Nsc) were established as in the schematic. (B) The protein of 4N was expressed in 293T cells and purified as described in the Methods section. (C) IF staining indicating the cellular localization of the recombinant proteins in U251 cells using His antibody (green). Bars, 10 μm. (D) Cell viability assay of the indicated cells was performed after treatment with Dox (.5 μM) for 48 h with or without 4N. Cell viability was evaluated by CCK‐8. (mean ± SD, *n* = 3, **p < 0.01). (E) Cell growth assay of U87 and U251 cells treated with 4N or Nsc. (mean ± SD, *n* = 3, **p < 0.01). (F) EdU incorporated assays of U87 and U251 cells treated with 4N or Nsc. The EdU labelled cells (red) were counted and the relative EdU positive cells were calculated. (mean ± SD, *n* = 5, **p < 0.01). (G) Double IF staining shows the differentiation of GBM3 CSCs after EGF withdrawal. Bars, 50 μm. (H) Western blot analysis of the expression of GFAP in GBM3 CSCs after EGF withdrawal. GAPDH served as a loading control. The right panel shows the statistical result. (mean ± SD, *n* = 3, **p < 0.01). (I) The BrdU‐ pulsed division assay in GBM3 CSCs. The right panel shows the quantitation of BrdU asymmetry or symmetry. **p < 0.01 (*χ* ^2^ test). Bars, 10 μm. (J) Schematic illustration of the evaluation of 4N as an optimization in TMZ sensitivity of CSCs in vivo. Mice were intracranially injected with GBM3 CSCs and subsequently received 10 intraperitoneal injections of DMSO or TMZ (40 mg/kg) with or without 4N from the 7^th^ day. Tumour volume was monitored by MRI at the 21^st^ day and animal survival times were recorded. (K) Representative tumour MRIs from each group. Quantitative analysis of the tumour size is shown in right (mean ± SD, *n* = 6, **p < 0 .01). (L) Kaplan–Meier survival curve is plotted. The lower panel shows the statistical results (*n* = 10).

The regulatory loop between TCF4N and p65 proposed that TCF4N could be an ideal target for GBM inhibition, especially in chemosensitivity facilitation. The dual function of TCF4N suggests that recombinant protein is a better option for tumour inhibition than gene transduction. Considering recombinant proteins do not readily enter cells, the amino acid of TCF4N was designed to be fused to a TAT peptide at the N‐terminal. TAT peptides can efficiently aid protein crossing biological membranes and promote the uptake of macromolecular cargo.^55^ The scrambled sequence of first 12 amino acids of TCF4N (Nsc) was designed as a control protein. To obtain a nuclear‐localization control protein, a tandem repeat of the nuclear localization signal (NLS) was fused at C‐terminus to induce nuclear translocation of Nsc (Figure 7A). The recombinant TCF4N protein (4N) was produced following eukaryotic expression system and purification by Ni‐NTA‐agarose columns (Figure 7B). Nsc was chemically synthesised directly. The subsequent IF staining indicated that 4N and Nsc protein could enter cells and localise to nuclei in U251 cells, while non‐nuclear‐forced Nsc protein (Nsc‐NLS^–^) were distributed in perinuclear region (Figure 7C). In terms of cellular viability, cell death was induced by Dox treatment and 4N spanning from 0–100 uM was added to examine whether it could promote the response to chemotherapy. As shown in Figure 7D, Nsc did not affect the cytotoxicity induced by Dox. On the contrary in 4N treated cells, cytotoxicity was strengthened in a 4N dose dependent manner, underlying a sensitizing promotion of 4N to chemotherapy. We noted that when the concentration of 4N reached 20 uM, its sensitivity‐promotion plateaued. Hence, this concentration was used to examine whether 4N works as endogenous TCF4N in GBM cells. As expected, cells treated with 4N showed higher growth capacity and proliferation rates than cells treated with vehicle or Nsc (Figure 7E,F). Subsequently, we determined whether the functions of 4N on GBM cells in vitro could be replicated in vivo. To this end, a low dose (TMZ/L, 10 mg/kg) and a high dose (TMZ/H, 40 mg/kg) of TMZ were used to evaluate whether 4N improves drug sensitivity. Mice were subcutaneously injected with U87 and received 10 intraperitoneal injections of DMSO or TMZ with or without 4N (10 ug) once every 2 days for 10 continuous injections from the 7^th^ day since implantation (Figure S8A). Tumour volume was measured every week and the tumour growth was expressed as fold change relative to the volume at 7^th^ day (Figure S8B). Comparing the tumour volume on the final experimental day, 4N administration resulted in a tumour promotion in vivo which corresponds to the in vitro tumour promotion effects. These results also showed that low dose TMZ failed to inhibit tumour growth, while combination with 4N resulted in a significant inhibition on tumour growth. Notably, 4N combined with either low or high dose TMZ resulted in tumour inhibition, suggesting that 4N can amplify tumour sensitivity to chemotherapy. To better document the effect of 4N in vivo, the tumours were collected on the 28^th^ day and the tumour weights were measured (Figure S8C). In addition to high dose TMZ therapy, the administration of 4N significantly promoted the anti‐cancer effect of TMZ/L, comparable to the effects of TMZ/H combined therapy (Figure S8D). Due to TCF4N overexpression‐mediated increase of GBM CSC differentiation, we next determined whether 4N could also be applied as an inhibitor for CSCs by assisting differentiation. GBM3 CSC spheroids were cultured in NSC medium without EGF to initiate differentiation. Double IF staining of GFAP and Nestin demonstrated the differentiation‐eliciting effect of 4N on GBM CSCs (Figure 7G), which was further confirmed by Western blot assay (Figure 7H). The balance of stem cell asymmetrical cell division (ACD) and symmetrical cell division (SCD) is important for normal life acidity. Accumulating evidence has suggested that increasing ACD of CSCs contributes to tumour initiation.^35^ Owing to the role of 4N in inducing differentiation of CSCs, we investigated the function in regulating SCD and ACD of GBM CSCs using a cell division assay reported previously.^35^ As shown in Figure 4, 7 administration increased ACD of CSLCs, suggesting its function on CSCs differentiation via promoting ACD. An important property of CSCs has been implicated in resistance to chemotherapy and radiotherapy.^56^, ^57^ We therefore asked whether 4N could increase the sensitivity of CSCs to TMZ. To document the inhibition of 4N on GBM CSCs in vivo, an intracranial orthotopic model was used in the following experiments. Mice were intracranially injected with GBM3 CSCs and subsequently received 10 intraperitoneal injections of DMSO or TMZ (40 mg/kg) with or without 4N from 7^th^ day post‐implantation. Tumour volume was monitored by Magnetic Resonance Imaging (MRI) on the 21^st^ day and animal survival times were recorded (Figure 7J). T2 weighted MRI imaging (Figure 7K) showed that the CSC‐bearing xenograft mice treated with TMZ displayed a moderate but significant reduction in overall tumour size after therapy. The involvement of 4N could improve the response of tumours to TMZ, thus inhibiting tumour growth to a greater extent. Kaplan–Meier analysis revealed that TMZ treatment resulted in a better survival outcome, while 4N administration greatly improved the expectation of survival (Figure 7L). Considering that inducing CSCs differentiation halts tumour growth and promotes response to therapies,^58^ 4N offers significant potential for tumour inhibition as a GBM CSC differentiation inducer and chemosensitiser.

## 4 DISCUSSION

**FIGURE 8:**
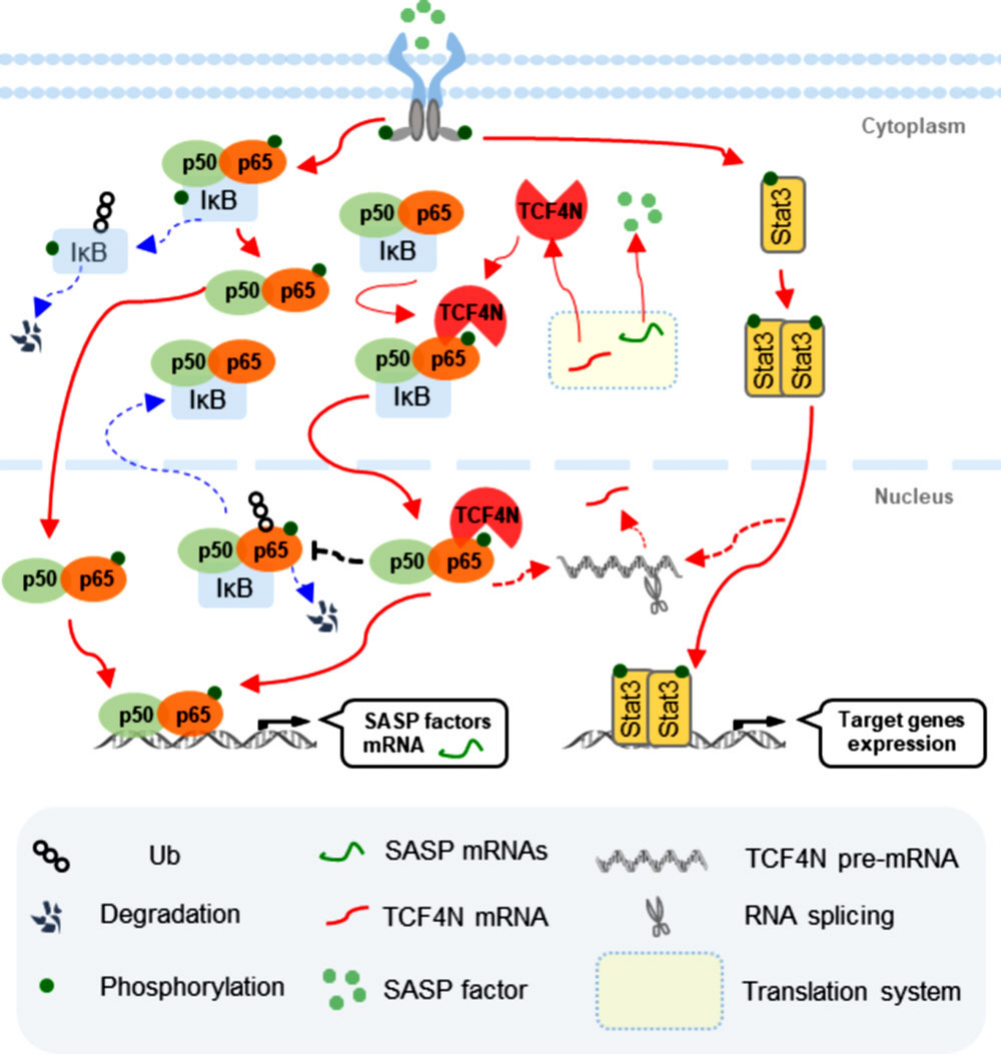
Molecular mechanism of TCF4N in the progression of GBM. Working model illustrating the regulation loop between p65 and TCF4N. TCF4N binds and promotes p65 S536 phosphorylation, nuclear‐translocation and stability, which ultimately upregulates NF‐κB target genes, including SASP factors; Feedback, nuclear p65 regulates the alternative splicing of *TCF7L2* pre‐mRNA, resulting in an increase of TCF4N.

TCF4N is a predicted target for the inhibition of tumourigenesis and for progression in cancers, operating by disrupting the interaction between β‐catenin and TCF4. While the present study disclosed that TCF4N possesses dual roles as a promoter and suppressor in GBM independent of β‐catenin. We provide evidence to position TCF4N as a regulator of NFκB/p65 and document the dual regulation of NFκB/p65 on GBM tumourigenesis and the chemotherapy response. This highlights a novel regulatory loop between TCF4N and p65 which promotes GBM tumourigenesis and chemosensitivity via S536 phosphorylation and nuclear‐translocation of p65 (Figure 8).

The downregulation of TCF4N predicts poor prognosis for GBM patients, suggesting that TCF4N may be a tumour suppressor of GBM. Functional studies showed that TCF4N is double‐faceted, promoting tumourigenesis and progression or enhancing tumour stem cell differentiation and chemosensitivity. Considering that patient tissues used in this study received standard TMZ chemotherapy, the observations explain the improved prediction of patients with higher TCF4N expression. It's interesting that TCF4N has both cancer‐promoting and anti‐cancer functions. Although containing β‐catenin‐binding capacity in hyperactive β‐catenin cancer cells, TCF4N has demonstrated to be a β‐catenin‐TCF4 independent regulator in GBM, which neither binds β‐catenin nor alters its cellular distribution and activation. This study also suggests that investigating TCF4 functions and regulatory mechanisms in cancers must account for the complexity caused by multiple isoforms.

A novel finding was that TCF4N overexpression increased IL6, IL8 and TNFα production. These molecules as well as other factors are major components of SASP. SASP may reinforce cell cycle arrest in senescent cells while also favouring tumourigenesis by enhancing cancer cell properties or promoting an immunosuppressive microenvironment.^59^ SASP's double sided functions in cancers are derived from divergent factors that participate in SASP or cellular senescence according to various environments.^60^ A typical feature of cellular senescence is a stable form of cell‐cycle exit. However, the present study failed to connect TCF4N to cellular senescence as TCF4N overexpression results in a robust cell proliferation under stress‐free conditions. Moreover, SASP factors commonly favour CSC maintenance and therapy resistance, thus SASP cannot explain the mechanism of TCF4N functioning in GBM. The components of SASP vary greatly across different tumours, cells and environments, thus there is no accurate definition for members of SASP which may explain its complex functions.^61^, ^62^ We speculate that the increase of SASP factors is just an accompanying phenomenon, rather than the primary mechanism of TCF4N actions. Moreover, SASP factors evoked by TCF4N can potentially promote tumourigenesis and therapy sensitivity,^62^, ^63^ which predominantly result from NF‐κB/p65 activation induced by TCF4N.^47^ NF‐κB/p65 and SASP are closely associated with the tumour immune microenvironment.^45^ While the immune microenvironment was beyond the scope of this study, it is conceivable that future study performed in the immune‐normal system could yield different results.

The most important finding of this study was the relationship between TCF4N and NF‐κB activation in GBM. It revealed that TCF4N induces p65 nuclear translocation and protects p65 from proteasome degradation by inhibiting ubiquitination. P65 is predominantly expressed in the cytoplasm, and is phosphorylated and translocated into the nucleus upon activation. The level of phosphorylation and nuclear expression of p65 is thus a marker of NF‐κB activity.^64^ As the central component of NF‐κB, p65 is also regulated at PTMs including ubiquitin–proteasome degradation.^48^, ^65^, ^66^, ^67^, ^68^ In our study, we identified TCF4N as a new binding protein that inhibits p65 ubiquitination and degradation in cells without an exogenous activator. We also provided evidence that the ubiquitination inhibition of TCF4N on p65 is dependent on direct binding. The finding that TCF4 feebly binds p65 in the nuclei but does not affect p65 expression and cellular distribution suggests that the action on p65 is unique to TCF4N. However, the mechanism of TCF4N‐induced activation and nuclear transport of p65 remains to be elucidated. The NF‐κB pathway is required for senescence, which in turn is accompanied by induction of SASP.^69^ Thus, the regulation loop between p65 and TCF4N may explain the mechanism by which TCF4N increases nuclear‐p65 stability and activation that subsequently induces SASP factor production and promotes further phosphorylation and nuclear transport of p65. Nevertheless, we cannot rule out the possibility that other molecular events occurring after TCF4N overexpression may also contribute to the activation of p65, such as the phosphorylation of IκB or other PTM regulators.

Since p65 is dispensable for TCF4N function in GBM, the core topic of this study was to document the dual roles of NF‐κB or p65 in GBM tumourigenesis and chemotherapy sensitivity. The de‐regulated NF‐κB signalling in cancers is widely linked to tumour progression, recurrence, poor survival, aggressiveness and chemoresistance, while the current study reinforces the notion that NF‐κB plays both pro‐oncogenic and anti‐oncogenic roles.^6^, ^70^ p65 was mainly expressed in the cytoplasm of glioma cells, indicating that p65 is not constitutively activated in gliomas. Although highly expressed in GBM, from gene/protein expression to in vitro and in vivo functional studies, the current study uncovered that overall p65 cannot be used as a prognostic indicator for GBM. Analysing the clinical relevance of p65 shows that patients with nuclear‐p65 expression have relatively better survival outcomes. Our functional experiments produce evidence that nuclear‐p65 contributes to the sensitivity of tumours to chemotherapy, highlighting nuclear‐p65 expression as a promising predictor of survival in GBM patients. The phosphorylation of S536 is associated with enhanced nuclear localization, decreased binding to co‐repressors, enhanced binding to co‐activators of p65 and elevated transcriptional activity. Recent findings postulate that the tumour restraining role of NF‐κB is associated with the phosphorylation of S536 and the induced apoptosis and senescence‐associated cytokine response.^6^, ^18^, ^19^ Here, we provide evidence that S536 phosphorylation endows p65 with pro‐survival and anti‐tumourigenic roles in GBM cells. Additionally, S536 phosphorylation induced p65 nuclear accumulation suggests that nuclear‐p65 is responsible for its anti‐cancer functions.^10^ The present study also observed a contradictory result that p65 overexpressing cells display different responses to chemotherapy between in vitro and in vivo environments. Based on the discovery that activated or nuclear p65 endows dual functions of NF‐κB in GBM, the different action of chemotherapy on p65 nuclear translocation/activation in vitro and in vivo likely explains the opposing results. Nevertheless, this result further documented that nuclear‐p65 is a determinant of the dual actions of NF‐κB in GBM.

The present study discloses a regulation loop between TCF4N and p65, providing a new insight that excessive p65 activation is favourable for chemotherapy in GBM. The current study demonstrates that a membrane‐penetrating peptide with TCF4N as the parent chain is a good option for promoting chemosensitivity. However, there is a potential risk that 4N‐induced p65 activation leads to endogenous TCF4N upregulation. Upon withdrawal of the chemotherapeutic drug, 4N‐induced TCF4N expression can be expected to restore its pro‐carcinogenic effect. Thus, an understanding of how p65 regulates the expression of TCF4N is necessary in order to develop strategies to inhibit the regulation of TCF4N by p65 upon 4N usage. Since various isoforms of TCF4 exist due to alternative splicing of *TCF7L2* pre‐mRNA, NF‐κB/p65 may regulate the splicing of pre‐mRNA but not at the transcriptional level. While no evidence has shown that p65 acts as an RNA‐binding protein, the regulation of p65 on TCF4N alternative splicing is likely indirect.

In conclusion, the current findings highlight key the importance of TCF4N as a novel regulator of NF‐κB through p65 interaction in GBM. It also provides a new target for GBM inhibition which can overcome the side effects of utilizing endogenous proteins. Additionally, disclosing the positive feedback loop between p65 and TCF4N may motivate future work to investigate the underlying mechanism.

## CONFLICT OF INTEREST

The authors have declared that no conflict of interest exists.

## Supporting information



Supporting information

Supporting information
