# Supplementary material for: The positive regulatory loop of TCF4N/p65 promotes glioblastoma tumourigenesis and chemosensitivity
Source: Clin Transl Med. 2022 Sep 18;12(9):e1042. doi: 10.1002/ctm2.1042 (PMC9482802; doi:10.1002/ctm2.1042)
Supplement: Supplementary file 1 — Supporting information [file CTM2-12-e1042-s002.docx]

**Supplementary Data 1. Clinical pathological information of GBM samples**

| **No.** | **Age** | **Gender** | **Disease specific survival in days** | **Overall survival (1=death, 0=alive)** | **△CT of TCF4N** | **IRS of cytoplamic-p65** | **IRS of nuclear-p65** |
| --- | --- | --- | --- | --- | --- | --- | --- |
| 1 | 57 | Male | 838 | 1 | 12.89 | 12 | 0 |
| 2 | 61 | Female | 1295 | 1 | 10.39 | 4 | 0 |
| 3 | 61 | Female | 838 | 1 | 10.20 | 3 | 1 |
| 4 | 49 | Male | 2695 | 1 | 12.13 | 3 | 1 |
| 5 | 81 | Male | 44 | 1 | 13.39 | 4 | 1 |
| 6 | 61 | Female | 1295 | 1 | 10.84 | 2 | 2 |
| 7 | 71 | Male | 930 | 1 | 11.66 | 4 | 2 |
| 8 | 50 | Female | 205 | 1 | 11.14 | 3 | 1 |
| 9 | 48 | Male | 33 | 1 | 14.02 | NA | NA |
| 10 | 50 | Male | 1022 | 1 | 14.36 | 6 | 1 |
| 11 | 47 | Female | 382 | 1 | 13.60 | 6 | 0 |
| 12 | 67 | Female | 48 | 1 | 10.44 | 6 | 0 |
| 13 | 56 | Female | 756 | 0 | 15.22 | 8 | 1 |
| 14 | 63 | Male | 229 | 1 | 12.57 | 8 | 2 |
| 15 | 64 | Female | 330 | 0 | 11.82 | 6 | 2 |
| 16 | 69 | Female | 288 | 0 | 13.96 | 3 | 2 |
| 17 | 32 | Female | 104 | 0 | 12.57 | 8 | 4 |
| 18 | 58 | Male | 76 | 0 | 13.24 | 6 | 1 |
| 19 | 66 | Male | 72 | 0 | 12.84 | 8 | 4 |
| 20 | 56 | Female | 61 | 0 | 15.22 | 2 | 1 |
| 21 | 66 | Female | 2240 | 1 | 10.93 | 8 | 0 |
| 22 | 58 | Male | 379 | 1 | 11.85 | 8 | 4 |
| 23 | 70 | Male | 791 | 1 | 17.59 | 6 | 1 |
| 24 | 42 | Female | 381 | 0 | 11.27 | 8 | 2 |
| 25 | 57 | Male | 874 | 1 | 14.21 | 4 | 1 |
| 26 | 58 | Female | 2360 | 1 | 12.32 | 4 | 2 |
| 27 | 68 | Male | 916 | 1 | 13.18 | 3 | 2 |
| 28 | 37 | Female | 255 | 1 | 16.89 | 8 | 0 |
| 29 | 73 | Male | 598 | 0 | 14.15 | 8 | 0 |
| 30 | 28 | Female | 1237 | 1 | 12.61 | 6 | 1 |
| 31 | 73 | Male | 598 | 1 | 12.92 | 8 | 0 |
| 32 | 60 | Female | 177 | 1 | 13.58 | 8 | 0 |
| 33 | 55 | Female | 899 | 1 | 13.35 | 4 | 0 |
| 34 | 69 | Male | 63 | 1 | 13.39 | 8 | 0 |
| 35 | 62 | Female | 727 | 0 | 15.31 | 3 | 0 |
| 36 | 52 | Female | 188 | 0 | 13.75 | 8 | 0 |
| 37 | 47 | Male | 779 | 1 | 12.93 | 8 | 0 |
| 38 | 40 | Male | 295 | 0 | 13.39 | 2 | 0 |
| 39 | 42 | Female | 336 | 1 | 13.64 | 4 | 0 |
| 40 | 52 | Female | 532 | 1 | 12.58 | 2 | 4 |
| 41 | 71 | Female | 373 | 1 | 13.36 | 8 | 0 |
| 42 | 57 | Male | 312 | 1 | 13.69 | 8 | 0 |
| 43 | 78 | Male | 1629 | 1 | 13.40 | 8 | 0 |
| 44 | 59 | Male | 838 | 1 | 14.04 | 2 | 0 |
| 45 | 60 | Male | 687 | 1 | 14.49 | NA | NA |
| 46 | 60 | Male | 657 | 1 | 14.20 | 8 | 0 |
| 47 | 54 | Male | 425 | 1 | 13.90 | 8 | 0 |
| 48 | 44 | Female | 2268 | 0 | 13.20 | 3 | 2 |
| 49 | 69 | Female | 149 | 1 | 12.83 | 8 | 0 |
| 50 | 69 | Female | 137 | 0 | 15.63 | NA | NA |

**Supplementary Data 2. Reference sequence for *TCF4N***

ATGCCGCAGCTGAACGGCGGTGGAGGGGATGACCTAGGCGCCAACGACGAACTGATTTCCTTCAAAGACGAGGGCGAACAGGAGGAGAAGAGCTCCGAAAACTCCTCGGCAGAGAGGGATTTAGCTGATGTCAAATCGTCTCTAGTCAATGAATCAGAAACGAATCAAAACAGCTCCTCCGATTCCGAGGCGGAAAGACGGCCTCCGCCTCGCTCCGAAAGTTTCCGAGACAAATCCCGGGAAAGTTTGGAAGAAGCGGCCAAGAGGCAAGATGGAGGGCTCTTTAAGGGGCCACCGTATCCCGGCTACCCCTTCATCATGATCCCCGACCTGACGAGCCCCTACCTCCCCAACGGATCGCTCTCGCCCACCGCCCGAACCCTCCATTTTCAGTCCGGCAGCACACATTACTCTGCGTACAAAACGATTGAACACCAGATTGCAGTTCAGTATCTCCAGATGAAATGGCCACTGCTTGATGTCCAGGCAGGGAGCCTCCAGAGTAGACAAGCCCTCAAGGATGCCCGGTCCCCATCACCGGCACACATTGTCTCTAACAAAGTGCCAGTGGTGCAGCACCCTCACCATGTCCACCCCCTCACGCCTCTTATCACGTACAGCAATGAACACTTCACGCCGGGAAACCCACCTCCACACTTACCAGCCGACGTAGACCCCAAAACAGGAATCCCACGGCCTCCGCACCCTCCAGATATATCCCCGTATTACCCACTATCGCCTGGCACCGTAGGACAAATCCCCCATCCGCTAGGATGGTTAGTACCACAGCAAGGTCAACCAGTGTACCCAATCACGACAGGAGGATTCAGACACCCCTACCCCACAGCTCTGACCGTCAATGCTTCCATGTCCAGGTGA

**Supplementary Table 1.** **Association between *TCF4N* expression and information of patients with GBM**

| Status | | *TCF4N* expression^#^, n | | Total | *p* value |
| --- | --- | --- | --- | --- | --- |
|  |  | LOW | HIGH | n |  |
| Gender | Male | 14 | 10 | 24 | 0.2575 |
|  | Female | 11 | 15 | 26 |  |
| Age | <55 yrs | 8 | 8 | 16 | >0.9999 |
|  | ≥55 yrs | 17 | 17 | 34 |  |
| Survival | ≤1 yrs | 12 | 8 | 20 | 0.0645 |
|  | 1-3 yrs | 12 | 10 | 22 |  |
|  | >3 yrs | 1 | 7 | 8 |  |

**p* values were analyzed by Chi-square test.

# According to the expression of *TCF4N*: the cutoff between LOW and HIGH was set at the median.

$ The total number of patients used in survival analysis was 50.

**Supplementary Table 2. Primers used for *TCF4N* DNA-seq**

| **Gene** | **Forward** | **Reverse** |
| --- | --- | --- |
| *P1* | CACACTTACCAGCCGACGTA | GAAGGCCCCGGTTCTTGGAA |
| *P2* | CAGGGAGCCTCCAGAGTAGA | GAAGGCCCCGGTTCTTGGAA |
| *P3* | CTCCACACTTACCAGCCGAC | GAAGGCCCCGGTTCTTGGA |
| *GAPDH* | AGAAGGCTGGGGCTCATTTG | AGGGGCCATCCACAGTCTTC |

**Supplementary Table 3. Primers used for qPCR**

| **Gene** | **Forward** | **Reverse** |
| --- | --- | --- |
| *TCF4N* | CCCCGTATTACCCACTATCGC | GGCCCCGGTTCTTGGAACTCA |
| *IL6* | AGACAGCCACTCACCTCTTCAG | TTCTGCCAGTGCCTCTTTGCTG |
| *IL8* | GAGAGTGATTGAGAGTGGACCAC | CACAACCCTCTGCACCCAGTTT |
| *TNFα* | CTCTTCTGCCTGCTGCACTTTG | ATGGGCTACAGGCTTGTCACTC |
| *GAPDH* | CAACTTTGGTATCGTGGAAGGACTC | AGGGATGATGTTCTGGAGAGCC |

**Supplementary Table 4. Sequence of sgRNA targeting *RELA***

| **sgRNA** | **Sequence** |
| --- | --- |
| sg1 | AGGGACAGTGCGCATCTCCC |
| Sg2 | ACTACGACCTGAATGCTGTG |
| Sg3 | GAGAAAGGACAGGCGGCAGG |
| NC | CGCTTCCGCGGCCCGTTCAA |

**Supplementary Table 5. PCR primers used for *TCF7L2* splicing**

| **Gene** | **Forward** | **Reverse** |
| --- | --- | --- |
| *TCF4N* | CCCCGTATTACCCACTATCGC | GGCCCCGGTTCTTGGAACTCA |
| *IL6* | AGACAGCCACTCACCTCTTCAG | TTCTGCCAGTGCCTCTTTGCTG |
| *IL8* | GAGAGTGATTGAGAGTGGACCAC | CACAACCCTCTGCACCCAGTTT |
| *TNFα* | CTCTTCTGCCTGCTGCACTTTG | ATGGGCTACAGGCTTGTCACTC |
| *GAPDH* | CAACTTTGGTATCGTGGAAGGACTC | AGGGATGATGTTCTGGAGAGCC |

**Supplementary Table 6. List of antibodies**

| Antigen | Primary Antibody | Dilution |
| --- | --- | --- |
| GAPDH | ThermoFisher; MA5-15738-1MG; mouse monoclonal | 1:2000 for WB |
| Lamin B1 | ABCAM; ab16048; rabbit polyclonal | 1:1000 for WB |
| Normal Rabbit IgG | Cell Signaling; 2729 | 1:100 for IP or ChIP; 1:500 for IHC or IF |
| Normal Mouse IgG | ABCAM; ab188776 | 1:100 for IP; 1:500 for IF |
| Nestin | Sigma; HPA007007; rabbit polyclonal | 1:2000 for WB; 1:500 for IF |
| GFAP | ABCAM; ab4648; mouse monoclonal | 1:2000 for WB; 1:50 for IF |
| BrdU | Sigma; B8434; mouse monoclonal | 1:200 for IF |
| β-catenin | Proteintech; 51067-2-AP; rabbit polyclonal | 1:1000 for WB; 1:100 for IF |
| active β-catenin | Cellsignaling; 19807; rabbit monoclonal | 1:1000 for WB |
| p65 | Cellsignaling; 8242; rabbit monoclonal | 1:1000 for WB; 1:400 for IF; 1:100 for IP; 1:30000 for IHC |
| p-p65 | Cellsignaling; 3033; rabbit monoclonal | 1:1000 for WB |
| Stat3 | Cell Signaling;9139; mouse monoclonal | 1:1000 for WB |
| p-Stat3 (S705) | Cell Signaling; 9145; rabbit monoclonal | 1:1000 for WB |
| Ubiquitin | ABCAM; ab7780; rabbit monoclonal | 1:1000 for WB |
| γH2A.X | Cell Signaling; 2577; rabbit monoclonal | 1:400 for IF |
| Ki67 | ABCAM; ab16667; rabbit polyclonal | 1:400 for IF |
| Flag-tag | Sigma; F7425; rabbit polyclonal | 1:2000 for WB; 1:200 for IP |
| HA-tag | Abmart; M20003; mouse monoclonal | 1:5000 for WB; 1:50 for IP; 1:2000 for IF |
| His-tag | Abmart; M30111; mouse monoclonal | 1:2000 for IF; 1:5000 for WB; |
