# Supplementary material for: The positive regulatory loop of TCF4N/p65 promotes glioblastoma tumourigenesis and chemosensitivity
Source: Clin Transl Med. 2022 Sep 18;12(9):e1042. doi: 10.1002/ctm2.1042 (PMC9482802; doi:10.1002/ctm2.1042)
Supplement: Supplementary file 2 — Supporting information [file CTM2-12-e1042-s001.docx]

**Supplementary Figure and Legends**

**
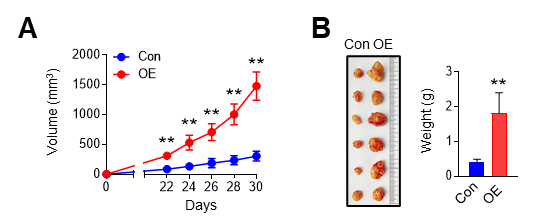
**

**Supplementary Figure 1.** **TCF4N promotes tumorigenesis *in vivo*.**

**A**. Growth curve of subcutaneous xenografts derived from indicated cells. The tumor volume was measured at the indicated days after implantation. (mean ± SD, n = 6, ***p* < 0.01).

**B**. Representative images of subcutaneous xenografts collected on the 30th day after implantation. The right panel shows the tumor weight analysis (mean ± SD, n = 6, ***p* < 0.01).

**
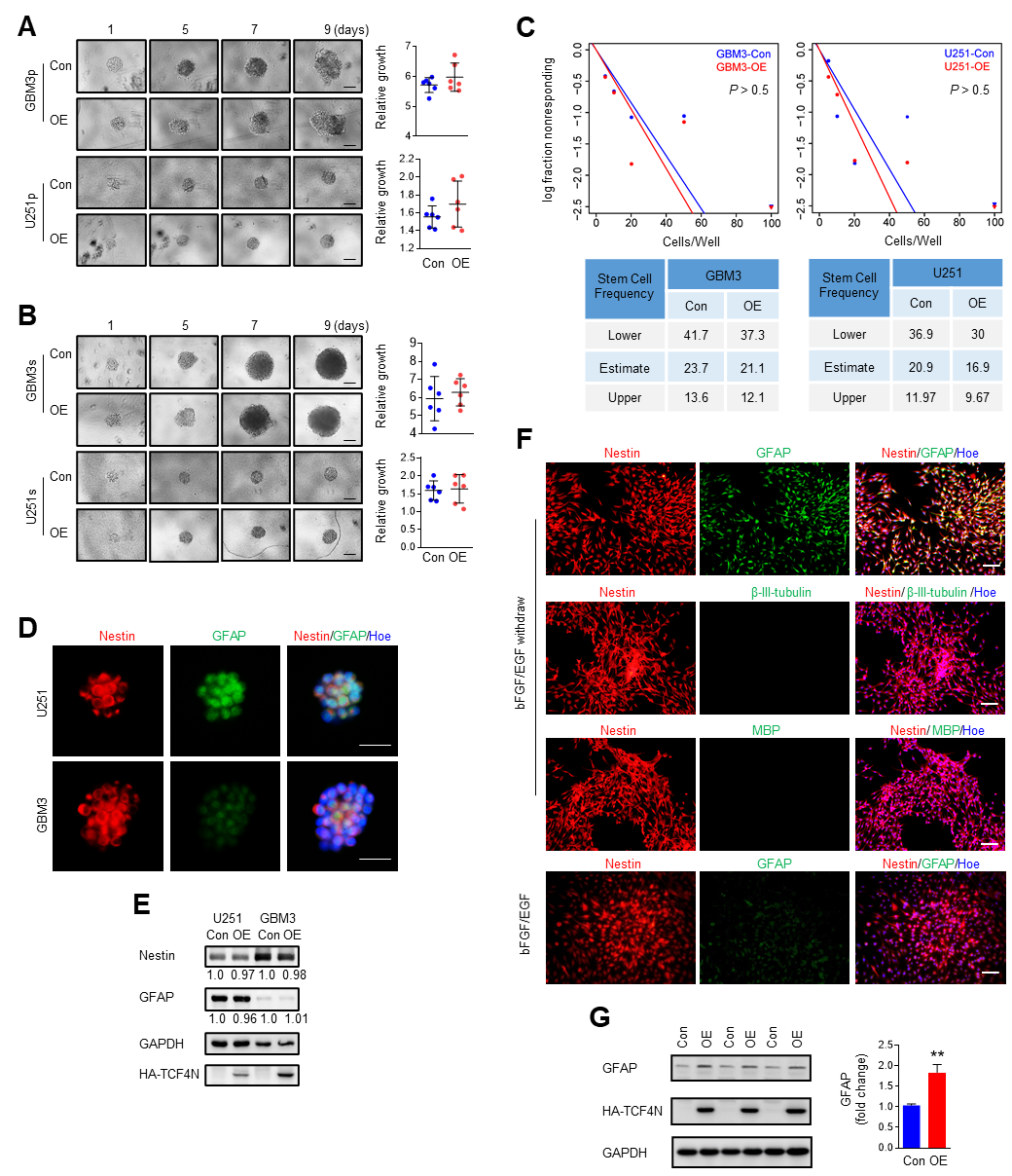
**

**Supplementary Figure 2. TCF4N promotes GBM stem cell differentiation.**

**A.** Primary spheroids growth assay of U251 and GBM3 cells. Cells were held in neuronal stem cell medium and allowed to form spheroids. Representative images show the single spheroids alteration over the indicated time. The right panel shows the relative spheroids growth from the 1^st^ day to the 9^th^. (mean ± SD, n=6). Bars, 200 μm.

**B.** Secondary passage of spheroids growth assay of U251 and GBM3 cells. The right panel shows the statistical results. (mean ± SD, n=6). Bars, 200 μm.

**C.** *In vitro* limiting dilution assays (LDAs) were performed to evaluate tumor spheres formation capacity.

**D.** Double immunofluorescence (IF) staining of Nestin and GFAP in indicated spheroids. Hoechst (Hoe) labeled nuclei. Bars, 50 μm.

**E.** Western blot analysis showing the expression of Nestin and GFAP in spheroids derived from the indicated cells. GAPDH served as a loading control. The relative expression of the indicated proteins is listed.

**F.** Double IF staining of Nestin and the indicated neural lineages markers are demonstrated in GBM3 spheroids after bFGF/EGF withdrawal. Bars, 100 μm.

**G.** Western blot analysis shows the increase of GFAP expression in GBM3 spheroids after bFGF/EGF withdrawal. GAPDH served as a loading control. The right panel shows the statistical results of GFAP. (mean ± SD, n = 3, ***p* < 0.01).


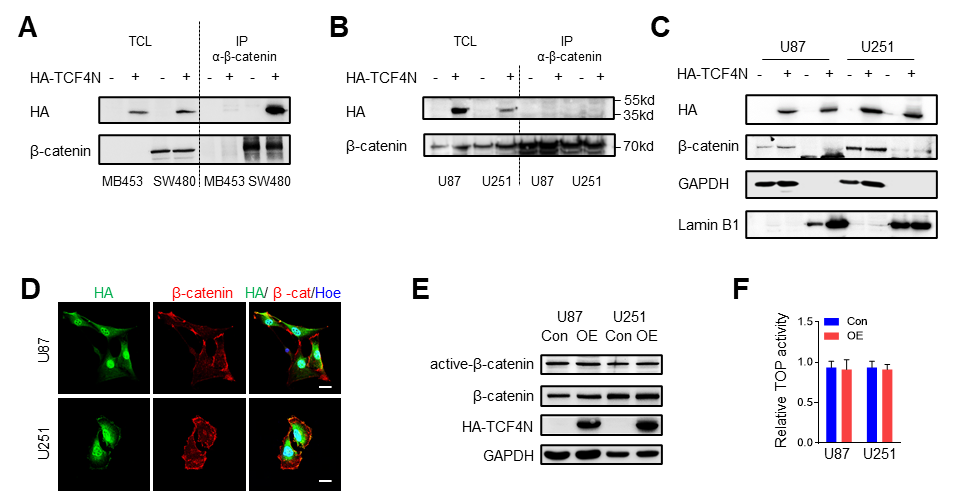


**Supplementary Figure 3. TCF4N functions in GBM via a Wnt/β-catenin independent manner.**

**A.** Immunoprecipitation (IP) analysis of TCF4N and β-catenin interaction. The indicated cells were transfected with HA-TCF4N plasmid and bound protein was immunoprecipitated by β-catenin antibody and detected by HA antibody. TCL, total cell lysate.

**B.** IP analysis of TCF4N and β-catenin interaction in GBM cell lines. Binding protein was immunoprecipitated by β-catenin antibody and detected by HA antibody.

**C.** Western blot analysis of cellular TCF4N and β-catenin expression in GBM cells. GAPDH and Lamin B1 served as cytoplasmic and nuclear loading controls, respectively.

**D.** Double IF staining showing the cellular localization of TCF4N and β-catenin in GBM cells. Bars, 10 μm.

**E.** Western blot analysis of the expression and activation of β-catenin in ectopic TCF4N expressing GBM cells. Active β-catenin was detected by non-phosphorylated β-Catenin (Ser33/37/Thr41) antibody. GAPDH served as a loading control.

**F.** Luciferase assay of TCF transcriptional activity in the indicated GBM cells using a TOPFlash luciferase reporter. (mean ± SD, n = 3).


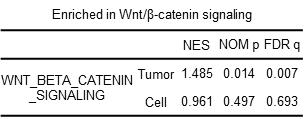


**Supplementary Figure 4. The enrichment of Wnt/β-catenin signaling gene set in tumors and cells.**


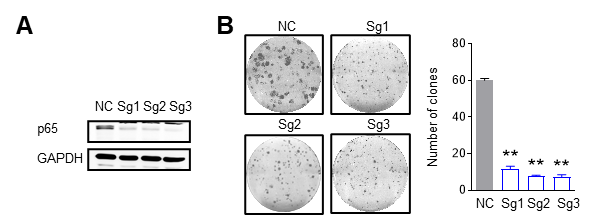


**Supplementary Figure 5. p65 knock-out inhibits colony formation of U87 cells.**

**A.** Western blot analysis of CRISPR/Cas9 mediated p65 knock-out. Sg, sgRNA sequence targeting *RELA*/p65.

**B**. Colony formation and growth of U87 cells with p65 knock-out (n=3, ***p* < 0.01)


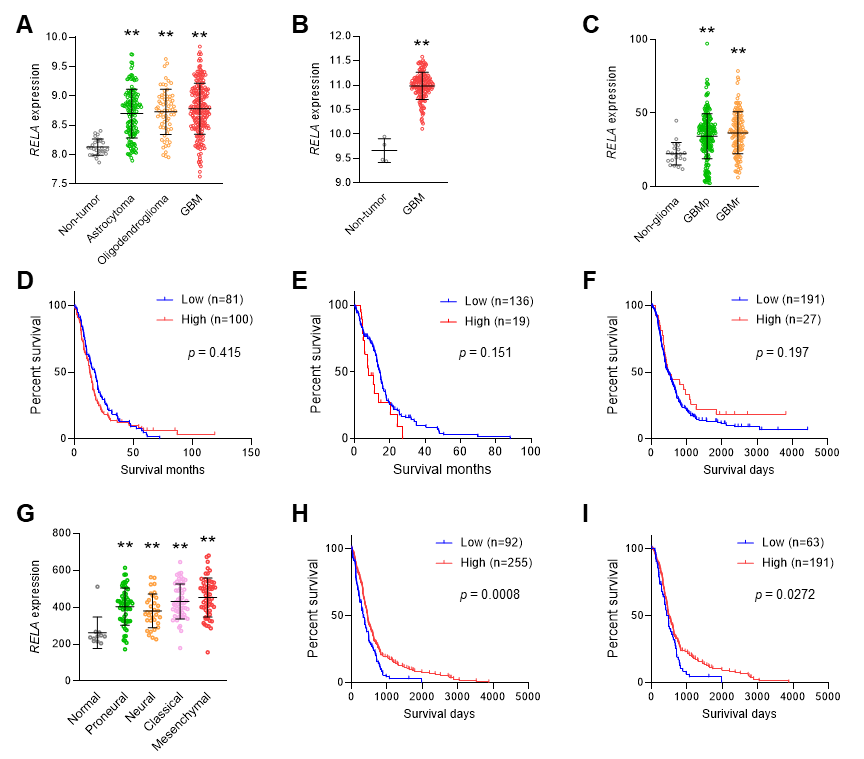


**Supplementary Figure 6. *RELA* is overexpressed in GBM.**

**A.** Rembrandt glioma dataset shows *RELA* is overexpressed in different histologic types of gliomas (***p* < 0.01).

**B.** TCGA-GBM RNAseq dataset shows *RELA* is overexpressed in GBM (***p* < 0.01).

**C.** CGGA-GBM RNAseq dataset shows *RELA* is overexpressed in primary (GBMp) and recurrent (GBMr) GBM (***p* < 0.01).

**D-F.** Overall survival analysis based on *RELA* expression in Rembrandt GBM dataset (D), TCGA-GBM RNAseq dataset (E) and CGGA-GBM RNAseq dataset (F) (Log-rank, *p* values indicated; Optimal cutoff).

**G.** TCGA-GBM dataset derived from Betastasis shows *RELA* is overexpressed in different subtypes of GBM (***p* < 0.01).

**H.** Overall survival analysis based on *RELA* expression in TCGA-GBM dataset derived from Betastasis (Log-rank, *p* values indicated; Optimal cutoff).

**I.** Overall survival analysis based on *RELA* expression in cases received chemotherapy TCGA-GBM dataset derived from Betastasis (Log-rank, *p* values indicated; Optimal cutoff).


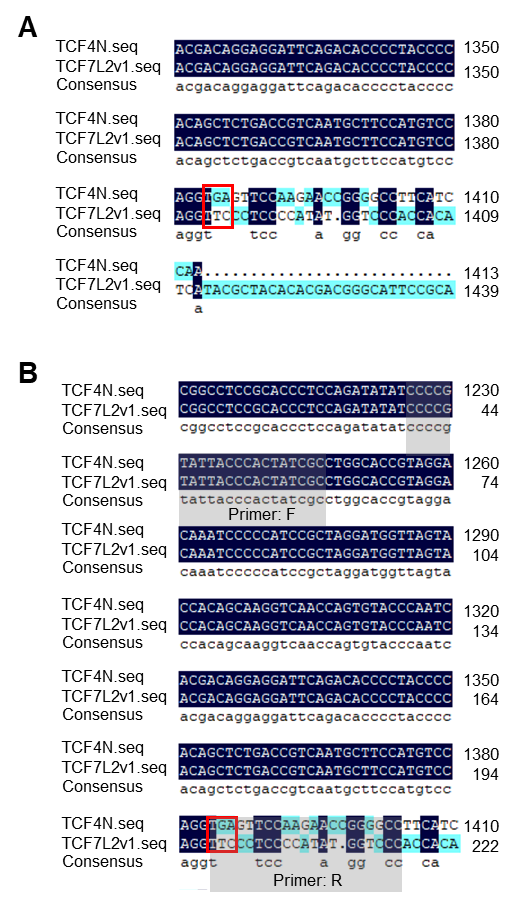


**Supplementary Figure 7. Quantitative PCR primers design for TCF4N.**

**A.** Nucleotide sequence alignment of *TCF4N* and *TCF7L2* isoforms v1. Red box shows a termination codon in *TCF4N* C-terminal.

**B.** The position of primers for quantitative PCR analysis of *TCF4N*.

**
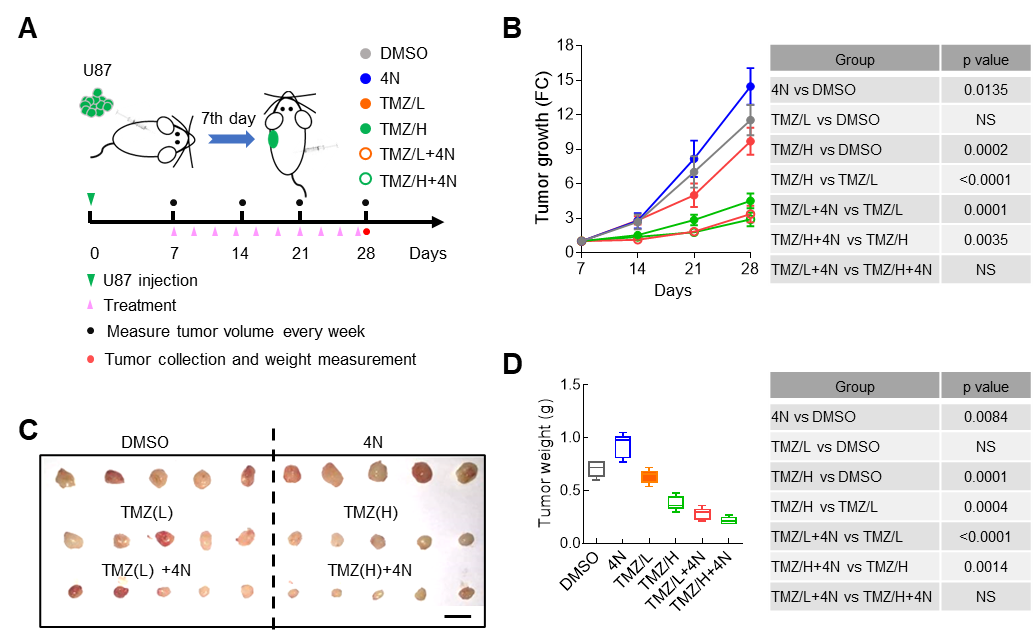
**

**Supplementary Figure 8. 4N increases tumor growth and chemosensitivity of GBM cells *in vivo*.**

**A.** Schematic illustration of the evaluation of *in vivo* tumor growth with the indicated treatment. Mice were intracranially injected with U87 cells and subsequently received 10 intraperitoneal injections of indicated treatment from the 7^th^ day post implantation. Tumors were collected at the 28^th^ day post implantation, tumor volume was measured as indicated.

**B.** Growth curve of subcutaneous xenografts derived from the indicated treatment. The statistical results of the tumor volume at final day are shown in the right panel. (mean ± SD, n = 5).

**C.** Representative images of subcutaneous xenografts collected at the 28^th^ day after treatment. Bars, 1 cm.

**D.** The statistical results of tumor weights. (mean ± SD, n=5).
